# Advanced biomimetic nanoreactor for specifically killing tumor cells through multi-enzyme cascade

**DOI:** 10.7150/thno.45456

**Published:** 2020-05-15

**Authors:** Wen Liu, Jinpei Wu, Xin Ji, Yandong Ma, Lamei Liu, Xiaoqing Zong, Haiyuan Yang, Jian Dai, Xiaoyuan Chen, Wei Xue

**Affiliations:** 1 Key Laboratory of Biomaterials of Guangdong Higher Education Institutes, Department of Biomedical Engineering, Jinan University, Guangzhou 510632, China; 2 Laboratory of Molecular Imaging and Nanomedicine, National Institute of Biomedical Imaging and Bioengineering, National Institutes of Health, Bethesda, MD 20892, USA; 3 Department of Orthopedics, The First Affiliated Hospital of Jinan University, Guangzhou 510632, China; 4 Institute of Life and Health Engineering, Key Laboratory of Functional Protein Research of Guangdong Higher Education Institutes, Jinan University, Guangzhou 510632, China

**Keywords:** biomimetic nanoreactor, multi-enzyme cascade, ROS modulation, β-lapachone, superoxide dismutase

## Abstract

Although the enzyme catalytic nanoreactors reported so far have achieved excellent therapeutic efficacy, how to accurately exert enzyme activity in the tumor microenvironment to specifically kill tumor cells and avoid systemic oxidative damage would be an inevitable challenge for catalytic nanomedicine. At the present study, we fabricate an advanced biomimetic nanoreactor, SOD-Fe^0^@Lapa-ZRF for tumor multi-enzyme cascade delivery that combined specifically killing tumor cells and protect cells from oxidative stress.

**Methods**: We first synthesized the FeNP-embedded SOD (SOD-Fe^0^) by reduction reaction using sodium borohydride. Next, SOD-Fe^0^ and Lapa cargo were encapsulated in ZIF-8 by self-assembly. In order to protect the cargo enzyme from digestion by protease and prolong blood circulating time, SOD-Fe^0^@Lapa-Z was further cloaked with RBC membrane and functionalized with folate targeting, resulting in the final advanced biomimetic nanoreactor SOD-Fe^0^@Lapa-ZRF.

**Results**: Once internalized, ZIF-8 achieves pH-triggered disassembly in weakly acidic tumor microenvironment. The released SOD-Fe^0^ and Lapa were further endocytosed by tumor cells and the Lapa produces superoxide anion (O_2_^-•^) through the catalysis of NQO1 that is overexpressed in tumor cells, while O_2_^-•^ is converted to H_2_O_2_ via SOD. At this time, the released ferrous ions from SOD-Fe^0^ and H_2_O_2_ are further transformed to highly toxic hydroxyl radicals (•OH) for specifically killing tumor cells, and there was no obvious toxicological response during long-term treatment. Importantly, SOD-Fe^0^@Lapa-ZRF enhanced the normal cell's anti-oxidation ability, and thus had little effect on the secretion of TNF-α, IL-6 and IL-1β pro-inflammatory cytokines, while effectively reversed the decreased activity of T-SOD and GSH-Px and remained stable MDA content after tumor treatment. *In vitro* and *in vivo* results indicate that the tumor microenvironment-responsive release multi-enzyme cascade have high tumor specificity and effective anti-tumor efficacy, and can protect cells from oxidative stress damage.

**Conclusion**: The biomimetic nanoreactor will have a great potential in cancer nanomedicine and provide a novel strategy to regulate oxidative stress.

## Introduction

Despite significant progress in cancer diagnosis and treatment, it remains one of the leading causes of death 1-3. Chemotherapy is the mainstay of clinical cancer treatment, but it is severely limited due to poor tumor selectivity and inevitable lethal side effects 4-6. Lactic acidosis of solid tumors in the tumor microenvironment (TME) and the subsequent metabolic reprogramming accelerate tumor growth, migration, and invasion 7-10. Therefore, the development of a drug delivery system (DDS) for acidic sensitivity of TME may be an alternative for improve the efficiency and selectivity of cancer therapeutics. In response to the acidic TME, chemodynamic therapy (CDT) relies on iron-based nanomaterials to dissolve ferrous ions under the mildly acidic TME and trigger Fenton reaction to catalyze hydrogen peroxide (H_2_O_2_) to generate highly toxic hydroxyl radicals (•OH), to achieve the purpose of killing tumor cells 11-13. In fact, various DDSs have been developed and designed for tumor CDT 14. However, one of the most critical challenges and obstacles is that due to tumor heterogeneity, the catalytic efficiency and selectivity of the delivered Fenton reagent are insufficient to achieve specific killing of tumor cells and minimal side effects on normal tissues 12. In addition, CDT will inevitably produce excessive metabolic by-products reactive oxygen species (ROS). The oxidative stress caused by these excessive ROS leads to various adverse physiological consequences such as tissue damage 15, cancer 16, diabetes 17, aging 18 and neurodegenerative diseases 19. Therefore, it is urgent to develop a new DDS that specifically kills tumor cells and enhances the ability of anti-oxidative damage in normal cells.

The intracellular superoxide dismutase (SOD) an endogenous antioxidant enzyme, can reduce the free radical level produced by oxidative stress in normal cells, and then exert anti-inflammatory and anti-apoptotic effects, which may dramatically weaken the damage of oxidative stress on normal cells 20. Studies have shown that comparison with cancerous analysis of normal tissues showed that SOD levels were significantly down-regulated in many types of malignant tumors, such as breast cancer, lung, and melanoma 21, 22. In addition, it has been found that increased expression of SOD can inhibit the malignant phenotypic progression of breast cancer cells and reduce the conversion of breast cancer cells to basal cells with the highest diffusion capacity 23-25. When SOD was transfected into melanoma cells, cell colonies could not form in soft agar and nude mice, reducing the proportion of tumorigenic cells 26. Therefore, overexpression of SOD can reduce tumor cell growth, metastasis, and other malignant features, which is a potential tumor suppressor 20. This suggests the possibility of targeting SOD as a tumor-specific treatment strategy. In addition, the intracellular superoxide dismutase (SOD) can catalyzes superoxide radicals (O_2_^-•^) to produce H_2_O_2_ and molecular oxygen (O_2_) 27, 28, where the accumulated H_2_O_2_ further converts downstream into •OH, which is ideal •OH generator 29-31. Therefore, introducing endogenous SOD into CDT as a •OH generator would be a multifunctional catalytic therapy strategy to specifically kill tumor cells by oxidative damage while protect normal tissues against oxidative stress, which would be remarkably attractive in cancer therapy.

Metal-organic framework (MOF) is an inorganic-organic porous composite formed by combining organic linkers with metal ion nodes, which has excellent biocompatibility, chemical stability and the capability of significantly tunable pore openings, and is used as basic building blocks for catalysis, sensors, biomedicine, etc 32-34. In recent years, MOF provides many benefits for enzyme immobilization, enhancing enzyme stability and maintaining enzyme activity 35, 36. However, the short blood half-life and lack of cancer targeting ability of MOF have severely hindered their medical application 37. Therefore, we introduced natural erythrocyte membranes into MOF nanoreactors of complex multi-enzyme cascades for designing more sensitive and stable advanced biomimetic nanoreactor.

**Scheme 1:**
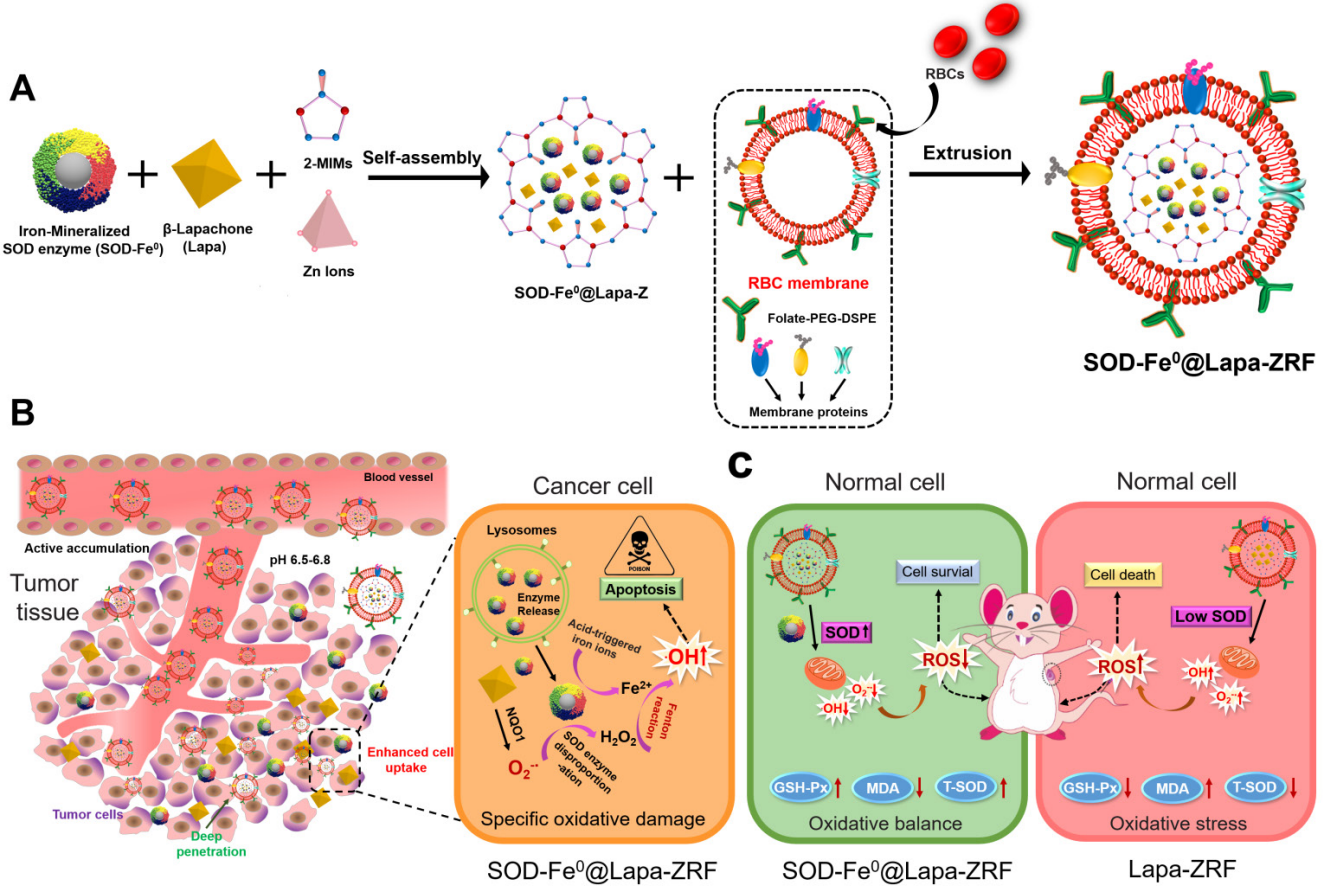
Schematic illustration of an Advanced Biomimetic Nanoreactor specifically killing tumor cells at the tumor tissue and its against oxidative stress in normal cells. (A) Synthetic procedure for SOD-Fe^0^@Lapa-ZRF nanoreactor. (B) After intravenous injection, SOD-Fe^0^@Lapa-ZRF are accumulated in tumor tissues and internalized by cancer cells. SOD-Fe^0^@Lapa-ZRF achieves pH-triggered disassembly in weakly acidic tumor microenvironment, resulting in rapid release of SOD-Fe^0^ and Lapa, and then produces a highly toxic •OH through a multi-enzyme cascade to specifically kill tumor cells. (C) In normal cells, SOD-Fe^0^@Lapa-ZRF adjusting the level of antioxidants and against oxidative stress and protect the body from oxidative stress damage. Lapa-ZRF causes oxidative stress *in vivo*.

Here, we designed pH-responsive biomimetic nanoreactor for tumor-specific CDT through multi-enzyme cascade. As shown in Scheme 1, the biomimetic nanoreactor SOD-Fe^0^@Lapa-ZRF utilized pH-responsive ZIF-8 as a stable support for the encapsulation of FeNP-embedded SOD (SOD-Fe^0^) and β-lapachone (Lapa), further followed by camouflaging with tumor targeting erythrocyte membrane. pH-responsive SOD-Fe^0^@Lapa-ZRF specifically kills tumor cells through multi-enzyme cascades, and could reduce oxidative damage to normal cells, avoiding undesired release of ROS. After internalization of tumor tissue, the nanoreactor degraded in tumor acidic microenvironment, resulting in rapid release of loaded SOD-Fe^0^ and Lapa and further endocytosis by the tumor cells. Lapa can produce sufficient O_2_**^-^**^•^ under the catalysis futile redox cycles of NAD(P)H:quinone oxidoreductase-1 (NQO1) enzyme, which is overexpressed 100-fold in tumor cells than normal cells 38-40. It has been found that overexpression of NQO1 in breast cancer cells promotes the invasion and/or metastasis related behaviors, which may be key to developing specific inhibitors that target NQO1 signaling 41-43. Then, the generated O_2_**^-^**^•^ was further translated to H_2_O_2_ through the SOD-mediated catalysis reaction. On the other hand, FeNP of SOD-Fe^0^ could be rapidly ionized in tumor acidic environment to release considerable amount of reactive ferrous ions Fe^2+^ in cancer cells. Subsequently, the released Fe^2+^ further catalyzed H_2_O_2_ to highly toxic hydroxyl radicals via a Fenton-like reaction 44, 45, resulting in oxidative damage of tumor cells, thereby specifically killing tumor cells. Moreover, FeNP cannot catalyze hydrogen peroxide under neutral pH conditions, thereby interrupting the cascade •OH generation, and SOD as an antioxidant enzyme has enhanced free radical scavenging ability, which helps reduce oxidative damage to normal cells. This multi-enzyme cascade biomimetic nanoreactor provides a promise strategy to developing tumor-specific CDT for the treatment of various malignancies and the regulation of prognostic oxidative stress.

## Results and Discussion

### Synthesis and Characterization of SOD-Fe^0^@Lapa-ZRF

**Figure 1:**
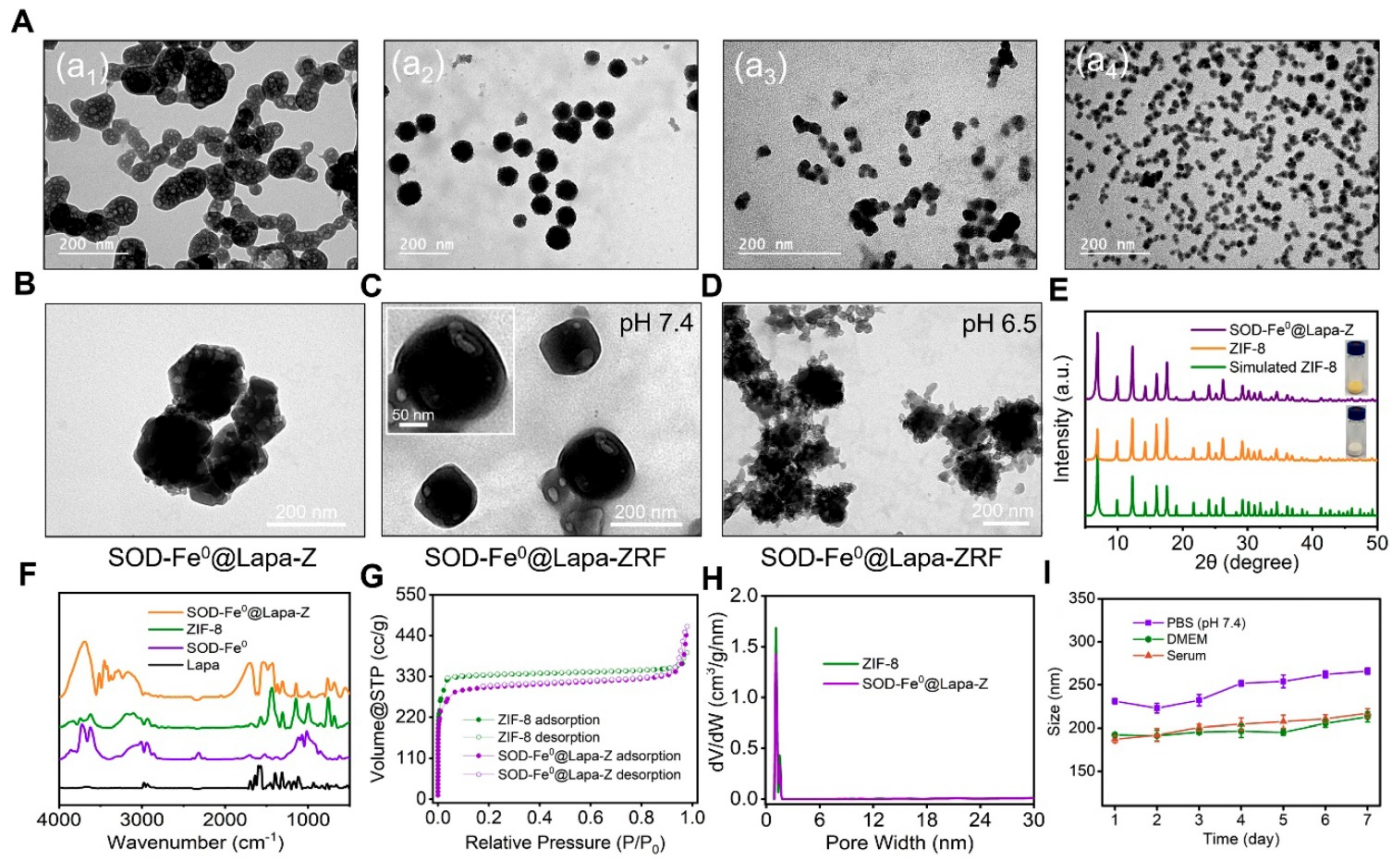
Characterizations of SOD-Fe^0^@Lapa-ZRF. (A) TEM image of SOD. TEM image of SOD-Fe under ammonium ferrous sulfate concentrations of (a2) 1.25 mmol, (a3) 0.62 mmol, and (a4) 0.31 mmol. TEM image of SOD-Fe^0^@Lapa-Z (B), and SOD-Fe^0^@Lapa-ZRF were incubated at pH 7.4 (C) and 6.5 (D). (E) XRD patterns of SOD-Fe^0^@Lapa-Z, ZIF-8 and Simulated ZIF-8. (F) FTIR spectra of SOD-Fe^0^@Lapa-Z, ZIF-8, SOD-Fe^0^ and Lapa. (G) Nitrogen sorption isotherms for ZIF-8 and SOD-Fe^0^@Lapa-Z. (H) The pore-size distributions of ZIF-8 and SOD-Fe^0^@Lapa-Z. (I) Sizes change of SOD-Fe^0^@Lapa-ZRF in PBS buffer, DMEM, and serum within 7 days.

According to previous reports, we first synthesized the FeNP-embedded SOD (SOD-Fe^0^) by reduction reaction using sodium borohydride. The morphology and diameter of SOD-Fe^0^ (Figure 1A) were optimized by adjusting the concentration proportion of SOD and ammonium ferrous sulfate. The hydrodynamic size of SOD-Fe^0^ was 18 nm (Figure 1a_4_ and S1a), and the Zeta potential of SOD-Fe^0^ was similar to that of the original SOD, (Figure S1b). The content of Fe in SOD-Fe^0^ was quantified by BCA protein assay kit and inductively coupled plasma mass spectrometry (ICP-MS). And the coupling amount of Fe was 277.8 μg/mg, thereby confirming that FeNP was successfully embedded in SOD. In addition, circular dichroism (CD) spectroscopy showed that the secondary structure of SOD in SOD-Fe^0^ was correlated with that of the original SOD (Figure S2), which indicated that SOD-Fe^0^ should have similar enzymatic activity as SOD enzyme. Next, SOD-Fe^0^ and Lapa were simultaneously immobilized in ZIF-8 by a co-precipitation procedure in aqueous solution. The transmission electron microscopy (TEM) (Figure S3a) and scanning electron microscope (SEM) (Figure S3b) images exhibited that the ZIF-8 had uniform internal structure and smooth surface. However, the TEM images of SOD-Fe^0^ and Lapa-coloaded ZIF-8 nanocomposite (SOD-Fe^0^@Lapa-Z) showed that the internal structure became heterogeneous (Figure 1B), while the surface got rougher (Figure S4). The average diameters of ZIF-8 and SOD-Fe^0^@Lapa-Z were 149 ± 7.8 nm and 176 ± 6.3 nm (Figure S5), respectively. And the zeta potentials were 22 and -6.1 mV (Figure S1b), respectively.

The crystal structure of SOD-Fe^0^@Lapa-Z and pure ZIF-8 was examined by X-ray diffraction (XRD) measurements (Figure 1E). The XRD patterns revealed that SOD-Fe^0^@Lapa-Z exhibited the space group *I*-43m in the overlapped XRD spectrum of pure ZIF-8. The Fourier transform infrared spectroscopy (FTIR) of SOD-Fe@Lapa-Z showed the adsorption bands at around 1650 and 1542 cm^-1^ which were attributed to amide I (C=O stretching mode) and amide II (combination between of N-H bending and C-N stretching modes) of SOD 46. And the peaks in the 1681-1810 cm^-1^ region and 1361 cm^-1^ was assigned to carbonyl groups (C=O) and methyl (C-H of -CH_3_) of Lapa (Figure 1F). Importantly, the loading efficiency of SOD-Fe^0^ and Lapa in the ZIF-8 were evaluated and reached up to 23% and 9.9%, respectively. These results suggested the successful encapsulation of SOD-Fe^0^ and Lapa in ZIF-8. In addition, the nitrogen adsorption/desorption study was performed to characterize the surface area and porosity of ZIF-8 and SOD-Fe^0^@Lapa-Z. As shown in Figure 1G and 1H, samples ZIF-8 and SOD-Fe^0^@Lapa-Z both displayed type I isotherms. However, the Brunauer-Emmett-Teller (BET) surface area of 1191.9 m^2^ g^-1^ for SOD-Fe@Lapa-Z was slightly less than that of ZIF-8 (1434.6 m^2^ g^-1^). Moreover, the corresponding pore volume of SOD-Fe@Lapa-Z (0.59 cm^3^ g^-1^) was lower than that of ZIF-8 (0.67 cm^3^ g^-1^). These data indicated that the encapsulated SOD-Fe^0^ and Lapa had slight influence to the porous structure of ZIF-8.

In order to protect the cargo enzyme from digestion by protease and prolong blood circulating time, SOD-Fe^0^@Lapa-Z was further cloaked with RBC membrane as previously reported 47, 48. This RBC membrane-cloaked SOD-Fe^0^@Lapa-Z (SOD-Fe^0^@Lapa-ZR) was further functionalized with folate targeting, resulting in the final advanced biomimetic nanoreactor SOD-Fe^0^@Lapa-ZRF. In contrast to SOD-Fe^0^@Lapa-Z (Figure 1B and Figure S4), SOD-Fe^0^@Lapa-ZRF showed an obvious core-shell structures under TEM with an average size of 190 ± 8.7 nm (Figure 1C and Figure S4). Upon the RBC membrane coating, it was found that the zeta potential of SOD-Fe^0^@Lapa-ZRF changed from -6.1 to -11.3 mV (Figure S1), comparable to that of SOD-Fe^0^@Lapa-Z. To further evidence the RBC camouflaging, RBC membranes and SOD-Fe^0^@Lapa-Z were labeled with Cy3 (red fluorescence) and FITC (green fluorescence), respectively. After that, their fusion was observed by confocal microscopy. The fluorescence images confirmed that the obvious overlap of yellow fluorescent signals derived from Cy3 and FITC in SOD-Fe^0^@Lapa-ZRF (Figure S6). These results indicated the successful RBC membrane cloaking on the surface of SOD-Fe^0^@Lapa-Z. Similarly, the TEM image also showed that the morphology of SOD-Fe^0^@Lapa-ZRF changed, the erythrocyte membrane ruptured and the nanoparticles were degraded at pH 6.5 (TME) (Figure 1D). In addition, we measured in vitro stability of SOD-Fe^0^@Lapa-ZRF under biological environment. As shown in Figure 1I, SOD-Fe^0^@Lapa-ZRF could maintain stable nanosize in phosphate-buffered saline (PBS) (pH 7.4) buffer, DMEM and serum solutions within 7 days, indicating the potential application of SOD-Fe^0^@Lapa-ZRF *in vivo*. These results indicated that SOD-Fe^0^@Lapa-ZRF undergo a significant dimensional change from physiological environment (pH 7.4) to tumor microenvironment (pH 6.5), indicating that SOD-Fe^0^@Lapa-ZRF has potential application value in tumor treatment.

### pH-Responsive Release of SOD-Fe^0^@Lapa-ZRF

**Figure 2:**
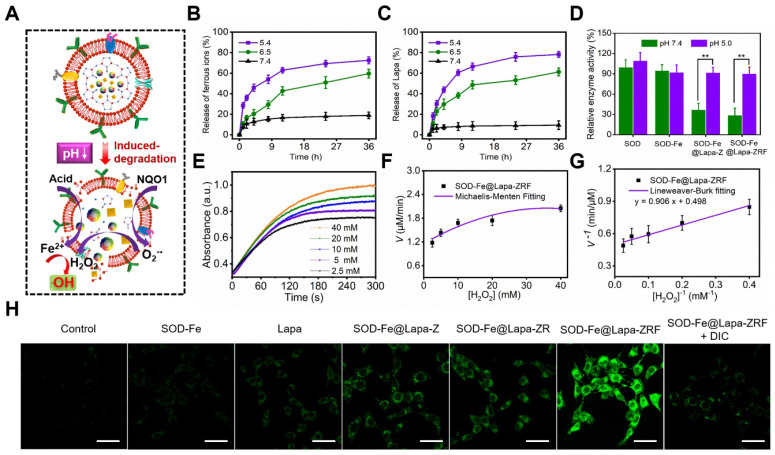
Multi-enzyme cascade reactions in SOD-Fe^0^@Lapa-ZRF. (A) Illustration of the multi-enzyme cascade biomimetic nanoreactor for catalysis. The ferrous ion (B) and Lapa (C) release from SOD-Fe^0^@Lapa-ZRF at various pH. (D) The relative enzymatic activity of SOD, SOD-Fe^0^, SOD-Fe^0^@Lapa-Z, and SOD-Fe^0^@Lapa-ZRF at various pH. (E) Time-course absorbance of SOD-Fe^0^@Lapa-ZRF at varied H_2_O_2_ concentrations. (F) Michaelis-Menten kinetics and (G) Lineweaver-Burk plotting of SOD-Fe^0^@Lapa-ZRF. (H) CLSM images of ROS levels in 4T1 cells treated with different agents without or with dicoumarol. The scale bar is 50 µm.

An ideal carrier should control release its cargo under specific conditions, such as low pH in the tumor microenvironment 49, 50. It is well known that metal-ligand bonds in ZIF-8 are stable under physiological conditions (pH ≈ 7.4), but are easily degraded under acidic conditions 51-53. To examine the effect of the pH-sensitive ZIF-8 on drug release of SOD-Fe^0^@Lapa-ZRF, the ferrous ions and Lapa release behaviors were measured at different pH. As shown in Figure 2B and C, there was strikingly slow release of ferrous ions and Lapa at pH 7.4, reaching up to 18.9% ferrous ions and 9.4% Lapa within 36 h. It indicated the excellent stability of the biomimetic nanoreactor. In contrast, the release rates of Fe^2+^ and Lapa at pH 6.5 reached 59.6 and 61.2% within 36 h, and that were 72.4 and 78.2% at pH 5.4 within 36 h, respectively. These results indicate that pH-induced decomposition of SOD-Fe^0^@Lapa-ZRF accelerated the release of ferrous ions and Lapa, demonstrating its potential to tumor-specific treatment application.

### The Cascade Catalytic Activity of SOD-Fe^0^@Lapa-ZRF

We investigated the multi-enzyme cascade catalytic activity of SOD-Fe^0^@Lapa-ZRF (Figure 2A). The 3, 3′, 5, 5′-tetramethylbenzidine (TMB) was used to measure the production of •OH to investigate the peroxidase-like activity of SOD-Fe^0^@Lapa-ZRF. The colorless TMB could be oxidized to chromogenic TMB cation radicals by Fe^2+^ in acidic condition, showing a strong absorbance of the oxidation product at 653 nm. Based on the aforementioned principles, the catalytic capability of Fe^2+^ in SOD-Fe^0^@Lapa-ZRF was investigated by using H_2_O_2_ and TMB as substrates through Michaelis-Menten steady-state kinetics (Figure 2E-G). According to the Lineweaver-Burke plotting, the Michaelis-Menten constant (*K*m) and maximum velocity (Vmax) were obtained. The Km and Vmax of SOD-Fe^0^@Lapa-ZRF were calculated to be 1.82 mM and 3.35 × 10^-8^ M s^-1^. Moreover, we also measured the capability of generating •OH of SOD-Fe^0^@Lapa-ZRF by the hydroxylation of terephthalic acid (TPA). From Figure S7, the TPA molecule mixed with the solution of SOD-Fe^0^@Lapa-ZRF and H_2_O_2_ showed the remarkably enhanced emission from TPA-OH at pH 5.4 and 6.5, relative to at pH 7.4. These results indicated that SOD-Fe^0^@Lapa-ZRF effectively produced •OH by Fenton reaction and exhibited peroxidase-like activity. In addition, we further evaluated the enzymatic activity of SOD in SOD-Fe^0^@Lapa-ZRF by using inhibiting pyrogallol autooxidation through UV-Vis spectroscopy 54. As shown in Figure 2D, the SOD within SOD-Fe^0^ retained 95.1 and 92.7% enzyme activity under pH 7.4 and pH 5.4, respectively, relative to the pure SOD. However, it is worth noting that the SOD in SOD-Fe^0^@Lapa-ZRF retained 29.1% and 90.5% of the enzyme activity of pure SOD at pH 7.4 and 5.4, respectively. These results indicated that under neutral condition, the stable encapsulation of SOD-Fe^0^ in ZIF-8 reduced the enzyme activity of SOD from SOD-Fe^0^@Lapa-ZRF, but with the pH-responsive release of SOD in the acidic condition, the excellent enzyme activity of SOD was re-triggered.

To further clarify the multi-enzyme cascade catalytic ability of SOD-Fe^0^@Lapa-ZRF, we subsequently examined the •OH generation efficiency in 4T1 breast cancer cells. 2′, 7′-dichlorofluorescein diacetate (DCFH-DA) as ROS detection probe was used, which could be rapidly oxidized to green fluorescence dichlorofluorescein (DCF). As shown in Figure S8, both SOD-Fe^0^@Lapa-Z and SOD-Fe^0^@Lapa-ZR effectively increased the ROS levels compared with the control, SOD-Fe^0^ and Lapa group. Moreover, SOD-Fe^0^@Lapa-ZRF induced the highest ROS level, rising to 268% ROS level at 120 min. However, the •OH generation by SOD-Fe^0^@Lapa-ZRF was significantly suppressed when the cells were pretreated with the NQO1 inhibitor of dicoumarol (DIC), decreasing to 152% ROS level within the same time. Meanwhile, the intracellular •OH generation was monitored using confocal laser scanning microscopy (CLSM) (Figure 2H). SOD-Fe^0^@Lapa-ZRF group exhibited more enhanced DCF fluorescence than the other groups. However, after adding DIC, SOD-Fe^0^@Lapa-ZRF group showed remarkable fluorescence decrease. The result further confirmed that SOD-Fe^0^@Lapa-ZRF selectively produced •OH in tumor cells via pH-responsive degradation and multi-enzyme catalytic cascades.

### Biocompatibility of SOD-Fe^0^@Lapa-ZRF

The excellent biocompatibility of nanocarriers is an important index for clinical medical applications 55-57. Previous studies have shown that the self-marker CD47 on the RBC surface can effectively inhibit macrophage phagocytosis by interacting with protein-alpha (SIRP-α) receptors 58, 59. Therefore, it was investigated whether RBC membrane cloaking had anti-phagocytosis ability by incubating RAW 264.7 murine macrophage-like cells with SOD-Fe^0^@Lapa-Z, SOD-Fe^0^@Lapa-ZR and SOD-Fe^0^@Lapa-ZRF. As shown in Figure S9a, the macrophage uptake rate of SOD-Fe^0^@Lapa-Z gradually increased, whereas the phagocytic capacity of SOD-Fe^0^@Lapa-ZR and SOD-Fe^0^@Lapa-ZRF decreased significantly, confirming that RBC membrane camouflage had a favorable ability to evade macrophage phagocytosis. Furthermore, the blood retention of SOD-Fe^0^@Lapa-ZRF was measured to assess their pharmacokinetic profile. As shown in Figure S9b, the blood concentration of SOD-Fe^0^@Lapa-Z was 4.43% ID g^-1^ (percentage injected dose per gram) 48 h postinjection. Notably, SOD-Fe^0^@Lapa-ZR and SOD-Fe^0^@Lapa-ZRF improved blood retention, showing approximately 12.9% and 13.3% of ID g^-1^ still in the blood circulation due to RBC membrane camouflage. These results indicated that the excellent blood circulation characteristics of SOD-Fe^0^@Lapa-ZRF provided a powerful guarantee for its in vivo application.

### Anti-inflammatory of SOD-Fe^0^@Lapa-ZRF

During the blood circulation, due to the non-specificity of the nanoparticles, they are easily recognized and phagocytized by normal cells, tissues and organs to cause allergy or hypersensitivity reactions and produce pro-inflammatory cytokines 12. Hence, the blood samples were collected within 72 h after i.v injection of different samples in female BALB/c mice, and the levels of the inflammatory cytokines including tumor necrosis factor alpha (TNF-α), interleukin-6 (IL-6) and Interleukin-1 beta (IL-1β) were detected by ELISA. As shown in Figure S10, compared with the control group, Lapa-Z significantly up-regulated the secretion of TNF-α, IL-6 and IL-1β pro-inflammatory cytokines, increasing by 108.6%, 204.5%, and 217.7%, respectively. Compared to Lapa-Z, SOD-Fe^0^@Lapa-Z showed less inflammatory response. Moreover, SOD-Fe^0^@Lapa-ZRF had little effect on the secretion of these three cytokines. This indicated that the camouflage of RBC membrane and the introduction of SOD-Fe^0^ reduced the interaction between Lapa-Z and normal cell, effectively avoiding the inflammatory response in vivo and avoids damage to normal tissues.

### Tumor Cellular Uptake of SOD-Fe^0^@Lapa-ZRF

**Figure 3:**
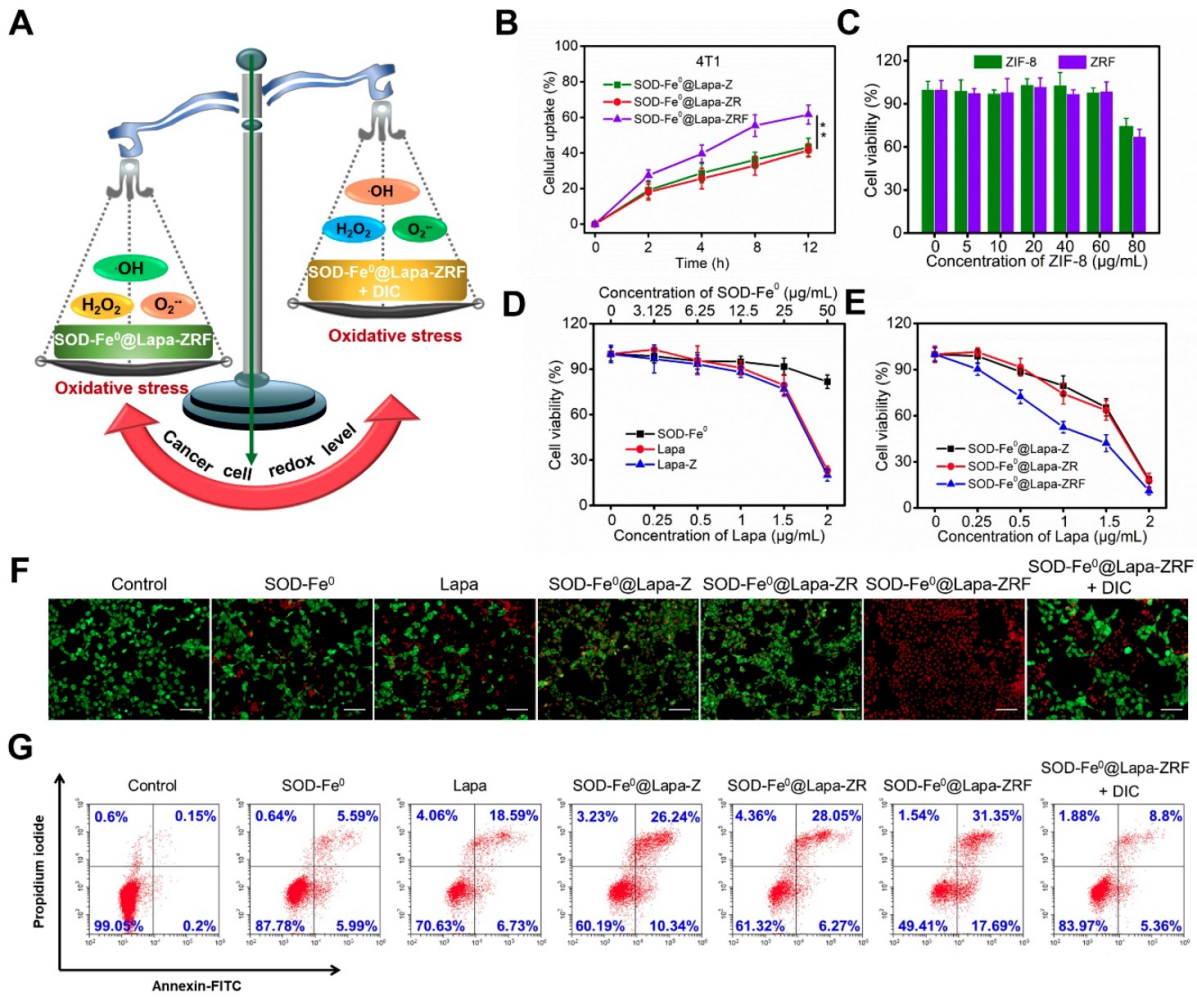
*In Vitro* Induced Oxidative Damage to Tumor Cells by SOD-Fe^0^@Lapa-ZRF. (A) Schematic illustration of cancer cells redox levels under the action of SOD-Fe^0^@Lapa-ZRF. (B) The cellular uptake of FITC-loaded SOD-Fe^0^@Lapa-Z, SOD-Fe^0^@Lapa-ZR, and SOD-Fe^0^@Lapa-ZRF in 4T1 cells. (C) 4T1 cells viability after treated with ZIF-8 and ZRF for 24 h. (D) 4T1 cells viability after incubation with SOD-Fe^0^, Lapa, and Lapa-Z for 24 h, as determined by CCK-8 assays. (E) Cytotoxic effect of SOD-Fe^0^@Lapa-Z, SOD-Fe^0^@Lapa-ZR, and SOD-Fe^0^@Lapa-ZRF on 4T1 cells. (F) CLSM images of calcein-AM/PI stained 4T1 cell after incubation with different agents. The scale bar is 100 μm. (G) Apoptosis analysis of 4T1 cells induced by SOD-Fe^0^, Lapa, SOD-Fe^0^@Lapa-Z, SOD-Fe^0^@Lapa-ZR, and SOD-Fe^0^@Lapa-ZRF with or without dicoumarol, and detected by flow cytometry. Data are shown as mean ± SD (n = 3). **p < 0.01.

We investigated the cellular uptake of FITC-labeled SOD-Fe^0^@Lapa-Z, SOD-Fe^0^@Lapa-ZR and SOD-Fe^ 0^@Lapa-ZRF in 4T1 cells. As shown in Figure 3B and Figure S11, SOD-Fe^0^@Lapa-ZRF with folate targeting exhibited higher cellular uptake in 4T1 than those of SOD-Fe^0^@Lapa-ZRF and SOD-Fe^0^@Lapa-Z. Moreover, the uptake of SOD-Fe^0^@Lapa-ZRF increased in a time-dependent manner. At 12 h, the cellular uptake of SOD-Fe^0^@Lapa-ZRF, SOD-Fe^0^@Lapa-ZR and SOD-Fe^0^@Lapa-Z were 61.6%, 41.5% and 43.3%, respectively. We further studied the retention time and distribution of SOD-Fe^0^@Lapa-ZRF in 4T1 cells by using confocal laser scanning microscopy (CLSM). Nuclei and lysosomes were fluorescently stained with Hoechst 33342 (blue) and Lyso Tracker (red). As shown in Figure S12, the colocalization of red and green fluorescence after treatment for 2 h indicated that the lysosome is the main intracellular target of SOD-Fe^0^@Lapa-ZRF. After 8 h of continuous internalization, most of green fluorescence from SOD-Fe^0^@Lapa-ZRF gradually penetrated into the whole cytoplasm through “proton sponge effect”. These results indicated that SOD-Fe^0^@Lapa-ZRF selectively uptakes 4T1 cells through receptor-mediated endocytosis.

### *In Vitro* Induced Oxidative Damage to Tumor Cells by SOD-Fe^0^@Lapa-ZRF

In vitro studies had demonstrated that SOD-Fe^0^@Lapa-ZRF efficiently produced •OH by multi-enzyme cascade catalytic reaction. Next, we investigated whether SOD-Fe^0^@Lapa-ZRF could specifically kill tumor cells by inducing tumor cell oxidative damage (Figure 3A). Hence, the cytotoxicity of different formulations in NQO1 overexpressing 4T1 cancer cells was investigated by using Cell Counting Kit-8 (CCK-8) assay. 4T1 cells were incubated with different concentrations of ZIF-8 and folate-modified red blood cell membrane-cloaked ZIF-8 (ZRF). As expected, ZIF-8 and ZRF did not show significant cytotoxicity to 4T1 cells within 24 h. Even at up to 80 μg/mL doses of ZIF-8, cell viability was still more than 65% (Figure 3C). Besides, the cell viability exhibited negligible decrease when cells were treated with SOD-Fe^0^ (Figure 3D). Similarly, with increasing concentrations of Lapa and Lapa-loaded ZIF-8 (Lapa-Z) (<1.5 μg/mL), no significant decrease of cell viability was observed. However, we found that SOD-Fe^0^@Lapa-Z and SOD-Fe^0^@Lapa-ZR showed dose-dependent toxicity to 4T1 cells with IC50 of 1.46 and 1.41 μg·mL^-1^, respectively, indicating that the cytotoxicity of SOD-Fe^0^@Lapa-Z and SOD-Fe^0^@Lapa-ZR on 4T1 cancer cells originated from oxidative damage of •OH by multiple enzyme cascades catalysis (Figure 3E). It is worth noting that the 4T1 cells treated with SOD-Fe^0^@Lapa-ZRF induced significantly higher cytotoxicity with an IC50 of only 0.91 μg mL^-1^, further confirming that the ability of SOD-Fe^0^@Lapa-ZRF to specifically kill tumor cells.

To observe the distribution of viable and dead cells, 4T1 cancer cells were stained with calcein AM (green) and propidium iodide (PI) (red) to identify live and dead/late apoptotic cells. As shown in Figure 3F, most cells of control group exhibited strong green fluorescence. However, much more red fluorescence was observed in SOD-Fe^0^@Lapa-Z and SOD-Fe^0^@Lapa-ZRF groups than that in SOD-Fe^0^ and Lapa groups. Furthermore, the SOD-Fe^0^@Lapa-ZRF group showed the most significant cytotoxicity, and the cell staining results were well matched to the cell viability assay results. Notably, the cell death was significantly reduced in the SOD-Fe^0^@Lapa-ZRF plus the NQO1 competitive inhibitor dicoumarol (DIC, 60 μM) group, which was attributed to the fact that DIC-induced NQO1 inhibition prevented Lapa to produce O_2_^-•^. And it further confirmed that NQO1 was essential for the production of •OH by multi-enzyme cascades. In addition, the cytotoxicity mechanism was examined by flow cytometry analysis using annexin V-FITC and propidium iodide (PI) double staining. The results showed that compared with the control, SOD-Fe^0^ and Lapa groups, the levels of apoptosis increased in the cells treated with SOD-Fe^0^@Lapa-Z and SOD-Fe^0^@Lapa-ZR, showing the apoptosis rates of 36.58 and 34.32% (Figure 3G), respectively. In contrast, the SOD-Fe^0^@Lapa-ZRF group induced the most significant apoptosis in 4T1 cells (49%). In addition, the introduction of DIC remarkably reduced the level of apoptosis in 4T1 cells, again indicating that SOD-Fe^0^@Lapa-ZRF selectively exerts therapeutic activity in tumor cells with high NQO1 expression through multi-enzyme cascade catalytic reaction.

### *In Vitro* Tumor Penetration of SOD-Fe^0^@Lapa-ZRF

Fibroblasts at the tumor site, as well as high concentrations of collagen, polysaccharides and other extracellular matrix environments are major obstacles to the diffusion of nanoparticles 60. Studies have shown that small size nanoparticles can improve tumor penetration capability. To assess the tumor penetration of SOD-Fe^0^@Lapa-ZRF, 4T1 cells multicellular tumor spheres (MCTS) were cultured to mimic solid tumors in vitro. Tumor spheres were treated with FITC-labeled SOD-Fe^0^@Lapa-ZR and SOD-Fe^0^@Lapa-ZRF for 12 h, and the penetration was observed by using CLSM. As shown in Figure S13a, at pH 7.4, the fluorescence signal of SOD-Fe^0^@Lapa-ZRF was mainly confined to the superficial MCTS. In contrast, under acidic conditions (pH 6.5), SOD-Fe^0^@Lapa-ZRF group showed significantly enhanced FITC fluorescence throughout the tumor sphere. However, the penetration of SOD-Fe^0^@Lapa-ZR was primarily localized at the edge of the tumor sphere at pH 6.5. This result demonstrated that tumor acid environment triggered degradation of the ZIF-8 effectively released the small nanosize SOD-Fe^0^ (18 nm), remarkably improving penetration ability.

### *In Vivo* Tumor Accumulation and Tumor Penetration of SOD-Fe^0^@Lapa-ZRF

As shown in Figure S13b, SOD-Fe^0^@Lapa-Z group exhibited relatively weak fluorescent signals in the tumor region. In contrast, the fluorescence signal of SOD-Fe^0^@Lapa-ZRF group in tumor tissue was stronger than the SOD-Fe^0^@Lapa-ZR group after 4 h postinjection, indicating the significant tumor targeting ability of SOD-Fe^0^@Lapa-ZRF. Then, mice were sacrificed at 24 h postinjection, and tumors and major organs were collected for ex vivo imaging. The fluorescent signal of SOD-Fe^0^@Lapa-ZRF distributed in tumor tissues was significantly higher than that of other organs (Figure S13c). Semiquantitative data showed that the fluorescence intensity of SOD-Fe^0^@Lapa-ZRF at tumor site was approximately 2.42- and 1.44-fold higher than that of SOD-Fe^0^@Lapa-Z and SOD-Fe^0^@Lapa-ZR (Figure S13d), respectively.

Following the promising results of tumor spheroids penetration and *in vivo* delivery, we further explored the ability of tumor deep penetration of SOD-Fe^0^@Lapa-ZRF. Tumor blood vessels and tumor hypoxic regions were stained with anti-CD31 and anti-HIF-1α antibodies. As shown in Figure S13e and S13f, the green fluorescence of SOD-Fe^0^@Lapa-ZRF spread more widely from tumor blood vessels than that of SOD-Fe^0^@Lapa-Z and SOD-Fe^0^@Lapa-ZR. The weak signals of SOD-Fe^0^@Lapa-Z and SOD-Fe^0^@Lapa-ZR were attributed to relatively low tumor accumulation. It is worth noting that the penetration ability of SOD-Fe^0^@Lapa-ZRF enhanced the potential to reach hypoxic areas after extravasation from the blood vessels, while the penetration depth of SOD-Fe^0^@Lapa-Z and SOD-Fe^0^@Lapa-ZR were limited. This was mainly due to that the degradation of SOD-Fe^0^@Lapa-ZRF into small nanosize SOD-Fe^0^ under acidic conditions, which significantly enhanced the accumulation of nanoparticle in farther distance from the tumor blood vessels and within the tumor hypoxia areas. These results confirmed that the size reduction of SOD-Fe^0^@Lapa-ZRF under slightly acidic tumor microenvironment had remarkable advantage to deliver SOD-Fe^0^ and Lapa to deep tumors, thereby increasing the efficiency of accurately exerting the enzyme activity at the tumor site.

### *In Vivo* Oxidative Damage Induced by of SOD-Fe^0^@Lapa-ZRF

**Figure 4:**
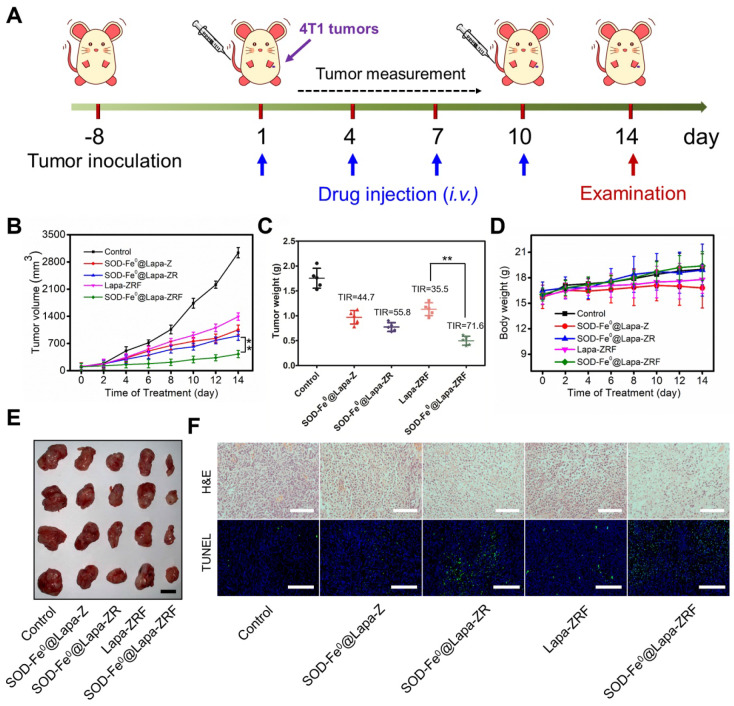
Oxidative Damage Induced by of SOD-Fe^0^@Lapa-ZRF in 4T1 tumor-bearing mice. (A) Schematic illustration of 4T1 tumor xenograft establishment and treatment process. (B) Tumor growth curves were monitored over the treatments. (C) Tumor weights of each group after treatment and tumor inhibition rate (IRT). (D) Body weight changes of mice during the treatment periods. (E) The representative photographs of resected tumors at the end of experiment. The scale bar is 5 mm. (F) Images of H&E staining and TUNEL assay of tumor sections after different treatments. The H&E scale bars represent 100 µm, The TUNEL scale bars represent 200 µm. Data are shown as mean ± SD (n = 3). **p < 0.01.

Inspired by excellent *in vitro* and *in vivo* performance, we further evaluated the antitumor activity of SOD-Fe^0^@Lapa-ZRF in 4T1 tumor xenograft model (Figure 4A). Once the tumor grew to 100 mm^3^, the BALB/c mice were randomly divided into 5 groups. The mice were intravenously injected with saline, SOD-Fe^0^@Lapa-Z, SOD-Fe^0^@Lapa-ZR, Lapa-ZRF or SOD-Fe^0^@Lapa-ZRF every 3 days (n = 5). The equivalent dose of SOD-Fe and Lapa was set to 2 mg kg^-1^ and 4.5 mg kg^-1^, respectively, in each injection. Compared with the control group, the mice injected with SOD-Fe^0^@Lapa-Z, SOD-Fe^0^@Lapa-ZR and Lapa-ZRF showed some therapeutic effects. Particularly, SOD-Fe^0^@Lapa-ZRF resulted in more pronounced inhibition of tumor growth than other groups (Figure 4D, 4E), which was mainly due to the specific oxidative damage induced by SOD-Fe^0^@Lapa-ZRF in tumor sites. Moreover, SOD-Fe^0^@Lapa-ZRF group (71.6% TIR) achieved a higher tumor inhibition rate (TIR) than that of Lapa-ZRF group (35.5% TIR), further emphasizing the important role of SOD-Fe^0^ in the multi-enzyme cascade catalysis (Figure 4C). Importantly, Lapa-ZRF treated mice showed weight loss compared to SOD-Fe^0^@Lapa-ZRF group, suggesting that SOD of SOD-Fe^0^@Lapa-ZRF could effectively reverse the potential systemic damage by oxidative stress (Figure 4D). In addition, necrosis and apoptosis analysis were performed by stained tumor sections with hematoxylin and eosin (H&E) and terminal deoxynucleotidyl transferase dUTP nick end labeling (TUNEL) (Figure 4F). Compared with the control group, the cell density in the tumor tissue was severely decreased after administration of SOD-Fe^0^@Lapa-ZRF, showing the typical apoptotic feature of severe vacuolization. Moreover, the TUNEL results also confirmed that SOD-Fe^0^@Lapa-ZRF induced a large number of cell apoptosis in tumor tissues compared to other nanoformulations. These results demonstrated that the multi-enzyme cascade biomimetic nanoreactor SOD-Fe^0^@Lapa-ZRF could accurately play a cascade catalytic role in the tumor microenvironment to specifically induce oxidative damage in 4T1 tumors.

### *In Vivo* Protection Effect

**Figure 5:**
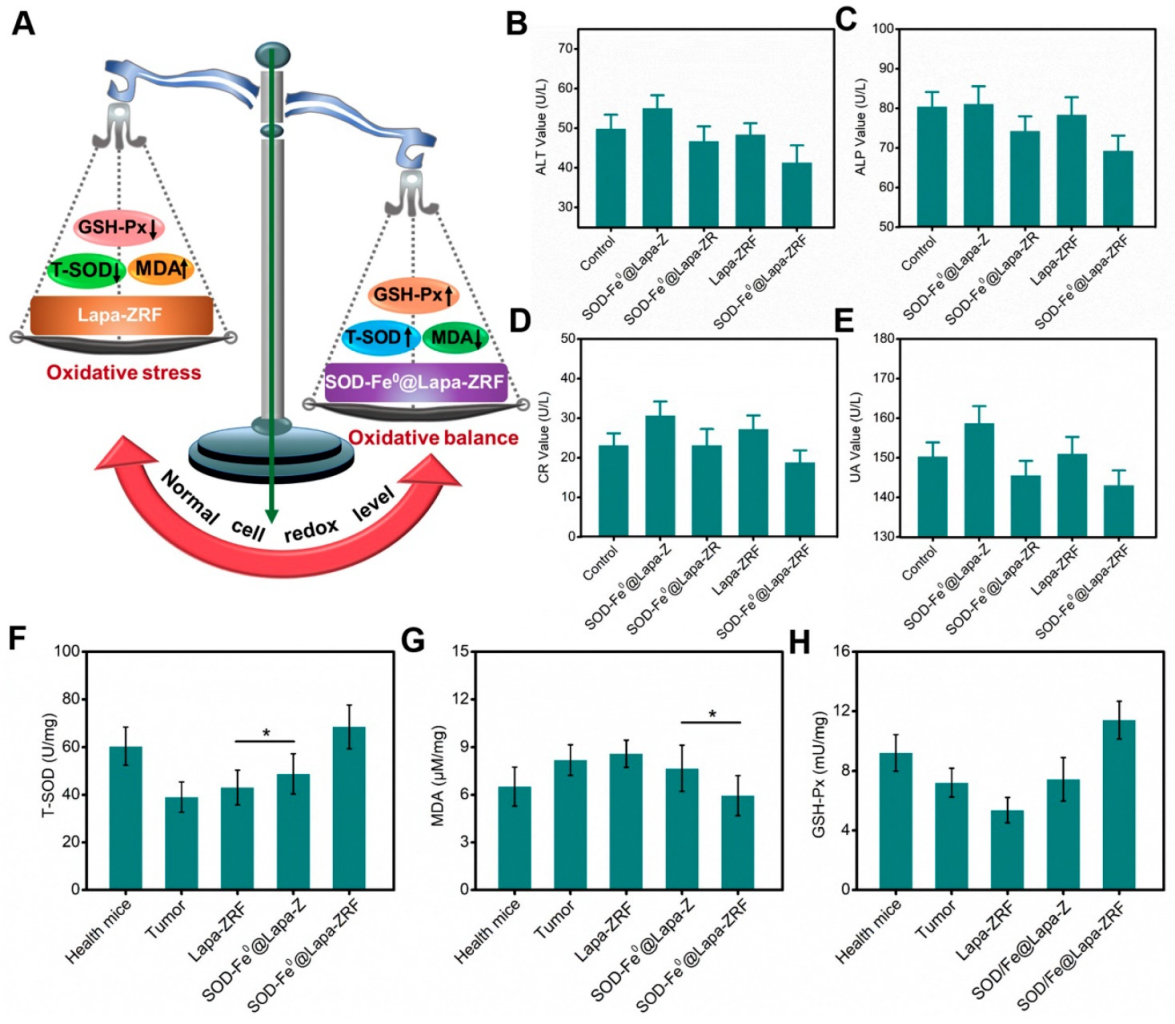
The prognostic against oxidative stress studies of SOD-Fe^0^@Lapa-ZRF. (A) Schematic illustration of normal cells redox levels under the action of SOD-Fe^0^@Lapa-ZRF. liver function index, ALT (B), ALP (C); and kidney function index, CR (D), UA (E). Antioxidant efficacy of SOD-Fe^0^@Lapa-ZRF on mice, (F) T-SOD, (G) MDA, and (H) GSH-Px. Data are shown as mean ± SD (n = 3). *p < 0.05, **p < 0.01 and **p < 0.01.

Although the enzyme catalytic nanoreactors reported so far have achieved excellent therapeutic efficacy, how to accurately exert enzyme activity in the tumor microenvironment and protect the body avoiding oxidative damage would be an inevitable challenge for catalytic medicine. At the present study, we fabricated an enzyme catalytic delivery system that could induced tumor oxidative damage through multi-enzyme cascade. Next, we investigated whether SOD-Fe^0^@Lapa-ZRF could against oxidative stress and protect normal cells from oxidative stress damage (Figure 5A). After all mice were euthanized, blood and primary organs were collected for blood biochemical and histological analysis to assess the systemic damage of the advanced biomimetic nanoreactor. As shown in Figure S14b, the white blood cell (WBC) and platelet (PLT) hematological indexes of mice treated with Lapa-ZRF showed a significant increase compared with SOD-Fe^0^@Lapa-ZRF group, indicating that certain inflammation and hematological toxicity in the mouse. Furthermore, as shown by the hepatic function markers (alanine aminotransferase (ALT), alkaline phosphatase (ALP), renal function markers (creatinine (CR), and uric acid (UA) indexes, SOD-Fe^0^@Lapa-ZRF could reverse liver and kidney damage caused by acute inflammation (Figure 5B-E). Moreover, in histological analysis, no obvious damage was found in the main organs of mice treated with SOD-Fe^0^@Lapa-ZRF (Figure S14a). The results showed that SOD-Fe^0^@Lapa-ZRF could effectively reverse the acute inflammation and liver and kidney damage caused by Lapa-ZRF after tumor treatment.

Moreover, we also monitored total superoxide dismutase (T-SOD), malondialdehyde (MDA, Lipid peroxide) and glutathione peroxidase (GSH-Px, Peroxidase) levels in the liver to evaluate the effect of SOD-Fe^0^@Lapa-ZRF to antioxidation indicators. Compared with healthy mice group (60.4 U mg^-1^ T-SOD and 9.22 mU mg^-1^ GSH-Px), the levels of T-SOD and GSH-Px in the liver of tumor-bearing mice decreased significantly (39.1 U mg^-1^ T-SOD and 7.22 mU mg^-1^ GSH-Px). This illustrated the high oxidative stress in tumor-bearing mice. As shown in Figure 5F and 5H, after different treatments, the T-SOD and GSH-Px levels in the Lapa-ZRF group were still low (43.1 U mg^-1^ T-SOD and 5.73 mU mg^-1^ GSH-Px), which could cause oxidative damage to the body. However, the SOD-Fe^0^@Lapa-ZRF group effectively reversed the decreased activity of T-SOD and GSH-Px (68.48 U mg^-1^ and 11.41 mU mg^-1^, respectively) after tumor treatment, which was higher than that in healthy mice. At the same time, compared with the healthy mice group, the MDA content remained stable without significantly fluctuate in the SOD-Fe^0^@Lapa-ZRF groups after treatment (Figure 5G). These results consistently indicated that SOD-Fe^0^@Lapa-ZRF will not cause damage to normal tissues, can effectively reduce oxidative stress in vivo and is an effective and safe strategy for cancer treatment.

### Specific tumor killing mechanism

We fabricate the advanced biomimetic nanoreactor, SOD-Fe@Lapa-ZRF for tumor specific multi-enzyme cascade delivery that combines the redox-function of SOD-Fe^0^ enzyme: oxidative damage in tumor tissue and anti-oxidation protection in normal tissue. Once internalized in tumor cell, the released Lapa produces O_2_^-•^ through the catalysis of NQO1, while O_2_^-•^ is converted to H_2_O_2_ via SOD. At this time, the released ferrous ions from SOD-Fe^0^ and hydrogen peroxide are further transformed to highly toxic •OH for cancer therapy. However, when the 4T1 tumor cells were pretreated with the NQO1 inhibitor of DIC, the •OH production by SOD-Fe^0^@Lapa-ZRF was significantly inhibited (Figure 2H), and cell death was significantly reduced (Figure 3F, G). This was mainly because the reduction of NQO1 hinders Lapa from producing O_2_**^-^**^•^, thereby inhibiting the multi-enzyme cascade. This indicated that SOD-Fe^0^@Lapa-ZRF can only produce •OH by multi-enzyme cascade and specifically kill tumor cells in tumor acidic environment with high expression of NQO1. However, in addition to low expression of NQO1 in normal tissue, FeNP cannot catalyze hydrogen peroxide under neutral pH conditions, thereby interrupting the cascade •OH generation, and SOD as an antioxidant enzyme has enhanced free radical scavenging ability, which helps reduce oxidative damage to normal cells, effectively reversing the decreased activity of T-SOD and GSH-Px. In short, SOD-Fe^0^@Lapa-ZRF showed excellent ability to specifically kill tumor cells, and to regulate oxidative stress and protect cells from oxidative stress damage.

## Conclusion

In summary, we have developed a biomimetic nanoreactor SOD-Fe^0^@Lapa-ZRF for specifically kill tumor cells via the multi-enzyme cascades, which generated a large number of •OH in the tumor microenvironment and could also increase the body's antioxidant index to against antioxidant stress. In acidic tumor environment, SOD-Fe^0^@Lapa-ZRF effectively disassembled, and the released enzyme and Lapa were further endocytosed by tumor cells. The released Lapa could increase the O_2_**^-^**^•^ levels in cancer cells by NQO1 catalysis, further converting O_2_**^-^**^•^ to H_2_O_2_ by SOD-mediated cascade reaction. Meantime, SOD-Fe^0^ could release ferrous ions in acidic intracellular environment. Subsequently, ferrous ions catalyzed H_2_O_2_ to decompose into highly toxic •OH in cancer cells through Fenton reaction, which specifically killing tumor cells, and there was no obvious toxicological response during long-term treatment. Importantly, SOD-Fe^0^@Lapa-ZRF enhanced the normal cell's anti-oxidation ability, and thus had little effect on the secretion of TNF-α, IL-6 and IL-1β pro-inflammatory cytokines, while effectively reversed the decreased activity of T-SOD and GSH-Px and remained stable MDA content after tumor treatment. The results in vitro and in vivo demonstrated that this multi-enzyme cascade biomimetic nanoreactor provided a novel approach to improve antitumor efficacy and protects normal cells from oxidative damage, exhibiting thus a great promise in future biomedical research.

## Experimental Section

### Materials

Zinc nitrate hexahydrate (Zn(NO_3_)_2_·6H_2_O) was supplied by Saen Chemical technology Co., Ltd. 2-methylimidazole (HMIM) was bought from Tokyo Chemical Industry Co., Ltd. β-Lapachone (Lapa) was purchased from Dalian Meilun Biotechnology Co., LTD. Folic acid-PEG-DSPE was obtained from ToYongBio Co., Ltd., Shanghai, China. Ammonium iron (II) sulfate and sodium borohydride (NaBH_4_) were purchased from Sigma-Aldrich Corp. Dicoumarol and terephthalic acid (TPA) was supplied by Shanghai Macklin Biochemical Co., Ltd. Superoxide dismutase (SOD) weas purchased from Shanghai Ryon Biological technology Co., Ltd. Sodium hydroxide and Dimethyl sulfoxide (DMSO) were purchased from Guangzhou Chemical Reagent Factory. Fluorescein isothiocyanate (FITC) was bought from Aladdin Industrial Co. Ltd. (Shanghai, China). The BCA protein assay kit and Hoechst 33342 were obtained from Invitrogen Corp. 2′,7′-dichlorofluorescein diacetate (DCFHDA) was bought from Sigma-Aldrich Corp. Cell Counting Kit-8 (CCK-8) was obtained from Dojindo Corp., Kumamoto, Japan.

### Synthesis of SOD-Fe^0^

Firstly, 30 ml aqueous solution of SOD (1 mL, 5 mg/mL) and ammonium iron (II) sulfate (0.125 mL, 25 mM) were mixed, and the reaction mixture stirred under N_2_ protection at room temperature for 0.5 h. Afterward, NaBH_4_ (0.25 mL, 50 mM) solution was slowly added to above reaction system under mechanical agitation at the speed of 0.1 ml min^-1^. The products were collected by centrifugation at 3000 rmp for 5 minutes and washed 3 times to remove the remnants.

### Synthesis of SOD-Fe^0^@Lapa-Z

1 mL SOD-Fe^0^ and 3 mg polyvinyl pyrrolidone (PVP) were dispersed in deionized water and stirred for 20 min to yield the PVP-modified SOD-Fe^0^. Then, 1 mL solution of PVP-modified SOD-Fe^0^ and 20 μL Lapa (1 mg) dissolved in DMSO were added to 0.5 mL of HMIM solution, and the mixture solution was magnetically stirred for 30 min. After that, 80 μL zinc nitrate solution (5 M) was slowly added into above mixture and stirred for another 20 min. After that, the as-synthesized products were collected by centrifugation at 10000 rpm for 10 min and then washed three times to remove the remnants.

### Synthesis of RBC Membrane and Folic acid inserted RBC membrane (RBC-Fa)

Briefly, fresh heparinized whole blood withdrawn from the female BALB/c mice (4-6 weeks) and subsequently centrifuged at 2500 rpm for 5 min at 4 °C to remove the plasma and the buffy coat. The collected RBCs were washed 3 times with ice-cold PBS and mixed with hypotonic lysing buffer (0.25 × PBS) for 4 h at 4 °C. After that, the released hemoglobin was removed by centrifuged for 3 times at 3500 g for 5 minutes at 4 °C. The resulting pink RBC ghost were collected after washing 3 times with PBS. To form RBC-Fa, 100 µL of the RBC membranes were incubated with 100 µg Folic acid-PEG-DSPE for 4 h at 4 ℃. The products of RBC-Fa were collected by centrifugation (3500 rpm) for 5 min and washed with water to remove free Folic acid-PEG-DSPE. Finaly, the RBC membrane and Folic acid inserted RBC membrane were stored in -80 °C for further use.

### Synthesis of SOD-Fe^0^@Lapa-ZR and SOD-Fe^0^@Lapa-ZRF

To encapsulate SOD-Fe^0^@Lapa-Z into RBC membrane or RBC-Fa, the vesicles derived of RBC or RBC-Fa were mixed with 1 mL of SOD-Fe^0^@Lapa-Z (2 mg/mL). the mixture was extruded through 200 nm polycarbonate porous membrane for at least 11 passes with an Avanti mini-extruder (AvantiPolar Lipids). The free RBC membrane or RBC-Fa were removed by centrifugation.

### Characterization

Transmission electron microscopy (TEM) and scanning electron microscopy images were observed by JEOL TEM-1210 operated under 120 kV and EX-250 SEM (horiba), Respectively. The phase of nanoparticles were recorded on a D8 Focus diffractometer (Bruker) with Cu Kα radiation (λ = 0.15405 nm). Fourier transform infrared (FTIR) spectra were examined on a Bruker VERTEX V70 spectrometer with the range of 500-4000 cm^-1^. Nitrogen (N_2_) adsorption desorption isotherms curves and the corresponding pore-size distributions were obtained using a TriStar 3000 (Micromeritics, Norcross, GA, USA) surface area analyzer. The size distribution and zeta potential was performed on a Zetasizer Nano ZS particle analyzer (Malvern Instruments Limited). The CD spectra of the samples were measured by a chirascan-plus spectropolarimeter (Applied Photophysics, UK). The fluorescence spectra were analyzed on a 970CRT spectrophotometer. The concerntration of Lapa were carried on high-performance liquid chromatography (HPLC, Agilent 1200, USA). HPLC was performed on a C18 column (Zorbax Eclipse XDB, 4.6 × 150 mm, 5 μm).

### pH-dependent Lapa and ferrous ion release

The *in vitro* drug release behaviors were performed at 37 °C with shaker at 100 rpm. SOD-Fe^0^@Lapa-ZRF were transferred into a dialysis bag (molecular weight cutoff = 3000 Da) and dialyzed in 10 mL of PBS solution (pH 7.4 or pH 5.4). At appropriate time points, 1 mL of the releasing buffer was taken out and an equal volume of fresh medium was returned. The released Lapa and ferrous ion were measured by HPLC and ICP-MS, respectively.

### Michaelis-Menten Kinetics Measurements

For a typical peroxidation reaction, different concentrations of H_2_O_2_ (2.5 mM, 5 mM, 10 mM 20 mM, and 40 mM) was mixed with TMB (1 mM) and SOD-Fe^0^@Lapa-ZRF (100 μg/mL) in sodium acetate (NaAc) buffer solution (20 mM pH = 5.2). The absorbance of the oxidization products (λ = 650 nm) using UV-vis spectrometer at 25 °C. The Michaelis-Menten kinetic curve of SOD-Fe^0^@Lapa-ZRF was obtained by plotting the initial velocity against H_2_O_2_ concentrations. The Michaelis-Menten constant (Km) and maximal velocity Vmax were calculated via the Lineweaver-Burk plotting:


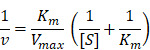


where *v* is the initial velocity, *[S]* is the concentrations of substrates.

### Hydroxyl radical (•OH) Generation

For terephthalic acid (TPA) oxidation, TPA (50 mM) solution was dissolved in NaOH (20 mM) solution. In a typical process, SOD-Fe^0^@Lapa-ZRF (0.3 mL, 100 μg/mL) was dispersed into H_2_O_2_ (20 mM) and TPA (5 mM) mixture solution (3 mL). After a few minutes, the fluorescence spectra of TPA-OH were monitored, and emission intensity of TPA-OH at 440 nm.

### SOD activity measurement

The SOD activity was assayed based on inhibition of pyrogallol autoxidation. In brief, 10 μL different concentrations of free SOD or SOD-Fe^0^@Lapa-ZRF was added into 10 μL pyrogallol (50 mM in 10 mM HCl) in Tris-HCl solution (4.5 mL, 50 mM, pH = 8.2). The absorbance intensity was measured at 325 nm by using UV spectroscopy to calculate the autoxidation rate. The activity of SOD in SOD-Fe^0^@Lapa-ZRF was then calculated from the specific inhibition rate compared with inhibition rate of free SOD.


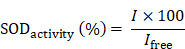


Where *I* and *I*_free_ are the inhibition rate of pyrogallol autoxidation in SOD-Fe^0^@Lapa-ZRF and free SOD, respectively.

### Cell culture

Murine macrophages (RAW264.7) were incubated in Dulbecco's modified Eagle's medium (DMEM) containing 10% FBS and 1% antibiotics (penicillin- streptomycin). 4T1 cells were incubated in RMPI 1640 medium containing 10% FBS and 1% antibiotics (penicillin- streptomycin). All cells were cultured at 37 °C in a humidified 5% CO_2_ atmosphere.

### *In vitro* cytotoxicity assay

4T1 cells were planted into 96-well plates (5000 cells per well) and cultured for 12 h for cytotoxicity assay. The 4T1 cells were incubated with ZIF-8, ZRF, Lapa, Lapa-Z, SOD-Fe^0^, SOD-Fe^0^@Lapa-Z, SOD-Fe^0^@Lapa-ZR and SOD-Fe^0^@Lapa-ZRF at different concentrations at 37°C. After 24 h incubation, the cells were washed twice with PBS and incubated in RMPI 1640 ontaining 10% CCK-8. The CCK-8 absorbance at 450 nm could measured using a microplate reader (MultiskanMk3, USA).

In order to intuitively observe the cell survival and death, the cell culture medium was removed. Then, these treated cells were stained with Calcein AM (AM) and Propidium Iodide (PI) for 20 min and subsequently the cell images were visualized using a microscope (Axio Observer A1, Zeiss, Germany).

### Cell Apoptosis Analysis

4T1 cells were seeded in a 6-well plate (1×10^5^ cells/well) for 12 h. The cells were then treated with SOD-Fe^0^ (25 μg/mL), Lapa (1.5 μg/mL of Lapa), SOD-Fe^0^@Lapa-Z (1.5 μg/mL of Lapa), SOD-Fe^0^@Lapa-ZR (1.5 μg/mL of Lapa), or SOD-Fe^0^@Lapa-ZRF (1.5 μg/mL of Lapa) with or without dicoumaral (60 μM) for 24 h. The medium was replaced with fresh medium and stained with Annexin V-FITC/PI according to the manufacture instructions (BD, USA). Finally, the apoptosis cells were examined by flow cytometer within 1 hour.

### Cellular Uptake and Intracellular Localization

4T1 or RAW264.7 cells were seeded in a 96-well plate (5000 cells/well) for 24 h. Then the cells were treated with SOD-Fe^0^@Lapa-Z, SOD-Fe^0^@Lapa-ZR, or SOD-Fe^0^@Lapa-ZRF labeled with FITC for 2, 4, 8, and 12 h. The cells were washed 3 times by PBS, and the fluorescence intensity of FITC was measured by fluorescence microplate reader with the excitation and emission wavelength at 488 and 525 nm, respectively. For intracellular location, 4T1 cells were planted in 2 cm confocal dishes and SOD-Fe^0^@Lapa-Z, SOD-Fe^0^@Lapa-ZR, or SOD-Fe^0^@Lapa-ZRF labeled with FITC was added at different time points. Then, the nucleus and was stained with Hoechst 33342 (blue) and lysosome was stained with lyso-tracker (red). Finally, the intracellular location of SOD-Fe^0^@Lapa-Z, SOD-Fe^0^@Lapa-ZR, or SOD-Fe^0^@Lapa-ZRF were observed by confocal laser scanning microscope (Zeiss LSM 880).

### Intracellular ROS detection

Intracellular ROS levels were measured using DCFH-DA. 4T1 cells were incubated with SOD-Fe^0^ (100 μg/mL), Lapa (8 μg/mL of Lapa), SOD-Fe^0^@Lapa-Z (8 μg/mL of Lapa), SOD-Fe^0^@Lapa-ZR (8 μg/mL of Lapa), or SOD-Fe^0^@Lapa-ZRF (8 μg/mL of Lapa) with or without dicoumaral (60 μM) for 6 h. Afterward, the cells were incubated with DCFH-DA (10 μM) for 30 min. Then, the production of intracellular ROS was detected at an excitation and emission wavelengths set at 488 and 525 nm, respectively.

Intracellular ROS observations on CLSM, 4T1 cells were seeded in 2cm confocal dishes at a density of 2×10^5^ cells/mL and incubated for 24 h. The cells were incubated with SOD-Fe^0^ (50 μg/mL), Lapa (8 μg/mL of Lapa), SOD-Fe^0^@Lapa-Z (8 μg/mL of Lapa), SOD-Fe^0^@Lapa-ZR (8 μg/mL of Lapa), or SOD-Fe^0^@Lapa-ZRF (8 μg/mL of Lapa) with or without dicoumaral (60 μM) for 6 h. Afterward, the cells were incubated with DCFH-DA (10 μM) for 30 min. Then, the cells were washed with PBS and stained with Hoechst 33258. The production of ROS was detected by CLSM.

### Penetration of SOD-Fe^0^@Lapa-ZRF in 4T1 Spheroid

The 4T1 cells (2 × 10^5^ cells/well) were seeded onto an ultralow adsorption 6-well plate and incubated for 48 h. The cells were incubated at 37 °C and culture medium was replaced every day. The 4T1 Spheroid formed spontaneously after 6 days. The 4T1 spheroid were treated with the FITC-loaded SOD-Fe^0^@Lapa-ZR and SOD-Fe^0^@Lapa-ZRF at different pH for 8 h. After that, the spheroids were washed with PBS and measured using *Z*-stack imaging from the top to the middle of spheroid by CLSM.

### *In Vivo* Tumor Penetration of SOD-Fe^0^@Lapa-ZRF

4T1 tumor-bearing BALB/c mice were randomly divided into four groups and administered with FITC--loaded SOD-Fe^0^@Lapa-Z, SOD-Fe^0^@Lapa-ZR, and SOD-Fe^0^@Lapa-ZRF (n = 3, each group) by the tumor reached 150-200 mm^3^. After 12 h injected intravenously, mice were sacrificed, and the tumors were harvested. Tumors of each group were fixed by paraformaldehyde and dehydrated in 30% sucrose for an additional 24 h. The tumor tissues were cut into 10 μm sections, and permeabilized by 0.1% Triton-X 100 for 20 min and blocked by 10% BSA for 2 h. After BSA blocking, the tumor sections were incubated overnight with anti-CD31 and anti-HIF-1α antibodies, respectively. Finally, the tumor sections were stained with DAPI and imaged with CLSM.

### *In Vivo* Pharmacokinetics

Female BALB/c mice bearing 4T1 tumor xenografts (n=3) received intravenous injected with of SOD-Fe^0^@Lapa-Z, SOD-Fe^0^@Lapa-ZR, and SOD-Fe^0^@Lapa-ZRF at a dose of 20 mg Zn kg^-1^. At different time points after the injection (1, 2, 4, 8, 12, 24, and 48 h), venous blood was collected from the tail veins. The Zn ions contents in blood were digested with chloroazotic acid and determined by ICP-MS.

### *In Vivo* Fluorescence Imaging

The female Balb/c mice with 4T1 tumors were performed in vivo imaging studies. When the tumor reached 100 mm^3^, the mice were intravenously injected with FITC-labeled SOD-Fe^0^@Lapa-Z, SOD-Fe^0^@Lapa-ZR, and SOD-Fe^0^@Lapa-ZRF (with equivalent Lapa of 2 mg kg^-1^). At 2, 6, 12, and 24 h after administration, and the fluorescent images were observed using VIS-FL imaging system (Cambridge Research & Instrumentation; Woburn, MA) at different time intervals. The mice were sacrificed 24 h post administration, and the tumors as well as normal tissues were harvested for ex vivo imaging. The region-of-interests were analyzed by using Living Image software.

### Immunogenicity of SOD-Fe^0^@Lapa-ZRF *In Vivo*

The female SD mice were randomly divided into four groups and intravenously injected with PBS, Lapa-Z, SOD-Fe^0^@Lapa-Z, and SOD-Fe^0^@Lapa-ZRF (with equivalent Lapa of 2 mg kg^-1^). At different time points after the injection (24, 36, and 72 h), venous blood was collected from the tail veins, and centrifuged to obtain plasma. The levels of TNF-α, IL-6, and IL-1β in plasma were performed by ELISA analysis.

### *In Vivo* Antitumor Efficacy

BALB/c nude mice bearing 4T1 tumors were randomly divided into four groups (n = 5) when the tumors size reached about 80-100 mm^3^. Then, the tumor mice were intravenously injected with saline, SOD-Fe^0^@Lapa-Z, SOD-Fe^0^@Lapa-ZR, Lapa-ZRF, and SOD-Fe^0^@Lapa-ZRF (equivalent to 2 mg/kg of Lapa) at 1, 4, 7, and 10 days. The tumor volume and body weight were recorded every 2 days for eight times. The tumor volumes (V) were calculated using the formula: V = W^2^ × L/2, where W and L represented tumor length and L represented tumor width, respectively. After observation for 14 days, the mice were sacrificed and tumors and main organs were collected and fixed with 4% paraformaldehyde and embedded in paraffin for analysis. At the same time, the blood of mices were collected to measure WBC and PLT. The blood samples were centrifuged to obtained plasma to measure the level of ALT, ALP, CR, and UA.

### Evaluation of Antioxidant Capacity* In Vivo*

Liver tissues were collected and then crushed via a ball grinding mill. Proteins were extracted from the liver tissues and the protein concentration was determined by BCA assay. The level of T-SOD, MDA, and GSH-Px vitality were evaluated using Total Superoxide Dismutase Assay Kit with WST-8, Lipid Peroxidation MDA Assay Kit, and Total Glutathione Peroxidase Assay Kit (Beyotime) according to the manufacturers' instructions, respectively.

### Statistical Analysis

All the experiments were carried out at least 3 times, and the data were presented as mean ± standard deviation. The statistical analysis was performed by two-tailed Student's t-test. Difference with *P < 0.05 or **P < 0.01 was considered statistically significant.
