# Supplementary material for: Advanced biomimetic nanoreactor for specifically killing tumor cells through multi-enzyme cascade
Source: Theranostics. 2020 May 15;10(14):6245–60. doi: 10.7150/thno.45456 (PMC7255035; doi:10.7150/thno.45456)
Supplement: Supplementary file 1 — Supplementary figures and tables. [file thnov10p6245s1.pdf]

Supporting Information

Advanced biomimetic nanoreactor for specifically killing tumor cells through multi-enzyme cascade

Wen Liu,[1,3] Jinpei Wu,[1] Xin Ji,[1] Yandong Ma,[1] Lamei Liu,[1] Xiaoqing Zong,[1] Haiyuan Yang,[1] Jian Dai,[1]* Xiaoyuan Chen,[2]* Wei Xue[1,3,4]*

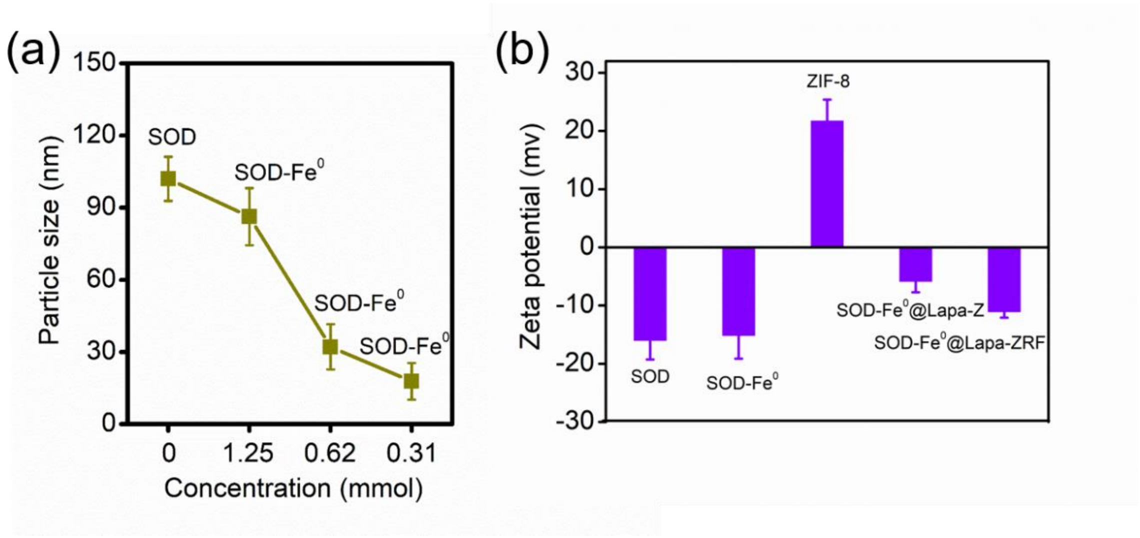

Figure S1. (a) The size of SOD and SOD-Fe^0 measured from Figure S1 TEM images. (b) Surface zeta potential of SOD, SOD-Fe^0, ZIF-8, SOD-Fe^0@Lapa-Z and SOD-Fe^0@Lapa-ZRF.

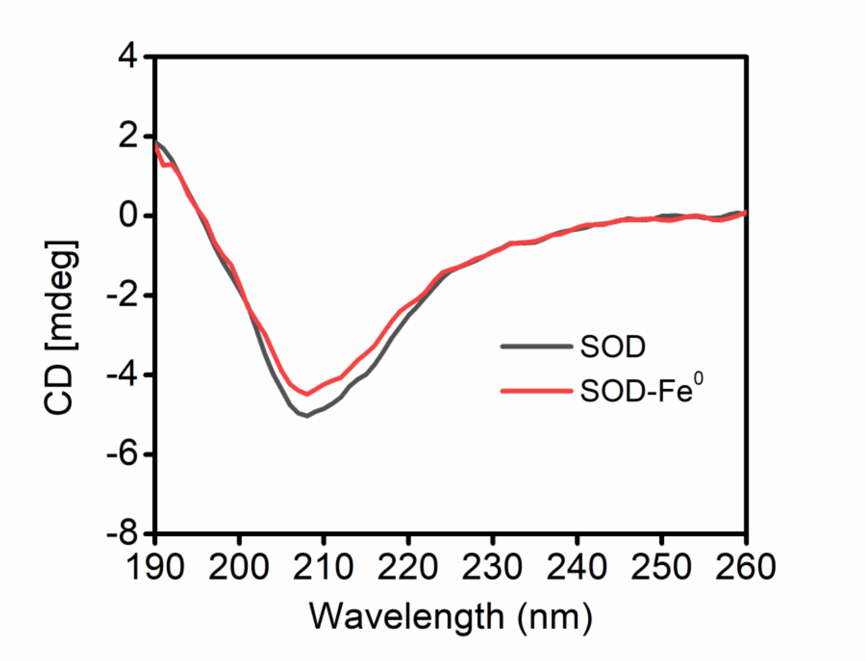

Figure S2. CD spectra of SOD and SOD-Fe^{0}.

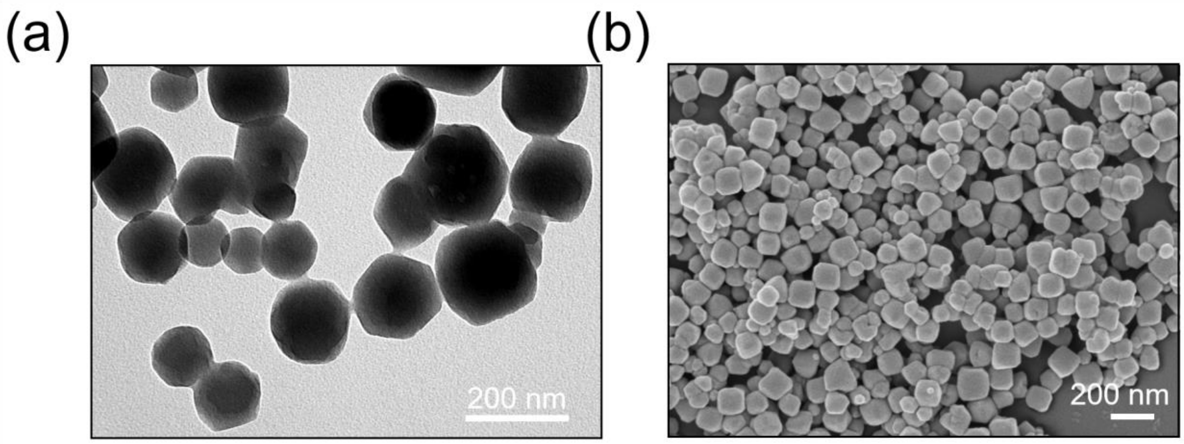

Figure S3. (a) TEM and (b) SEM image of ZIF-8.

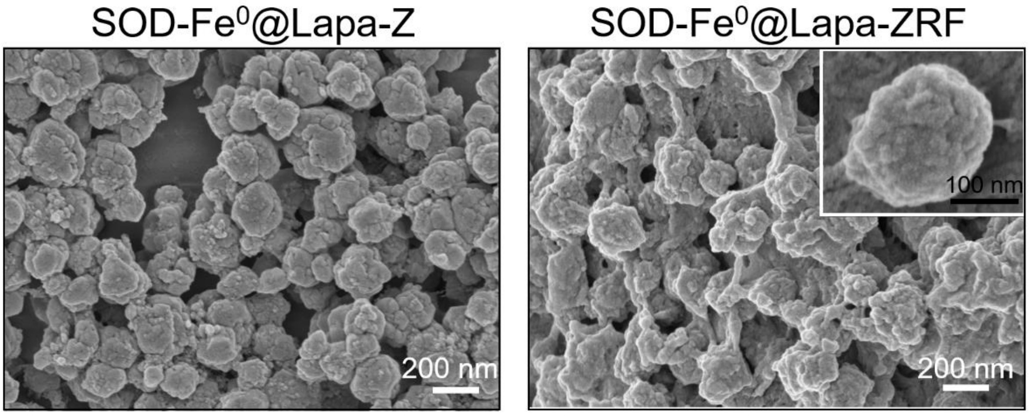

Figure S4. SEM image of SOD-Fe^0@Lapa-Z and SOD-Fe^0@Lapa-ZRF.

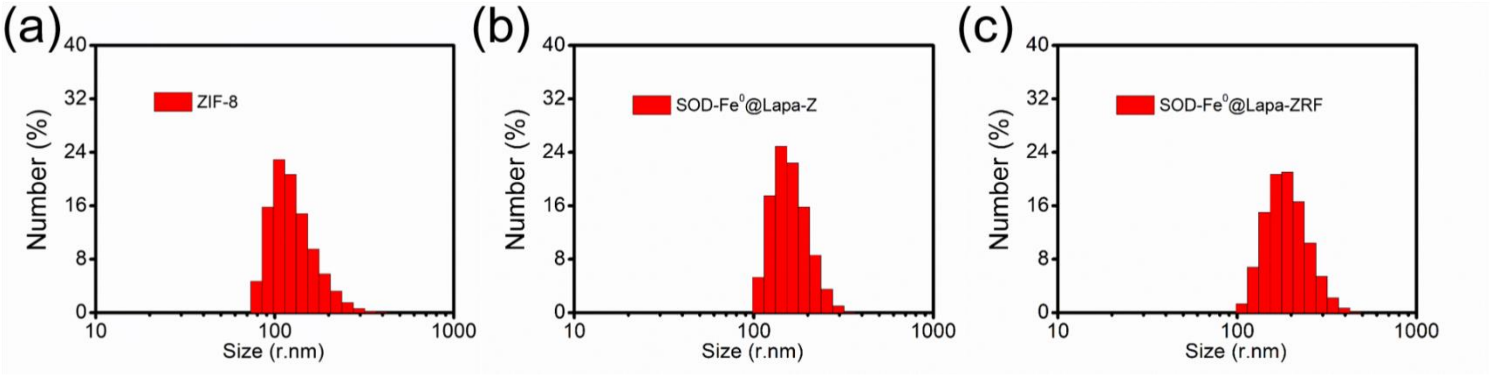

Figure S5. Hydrodynamic size distribution of ZIF-8 (a), SOD-Fe^0@Lapa-Z (b) and SOD-Fe^0@Lapa-ZRF (c).

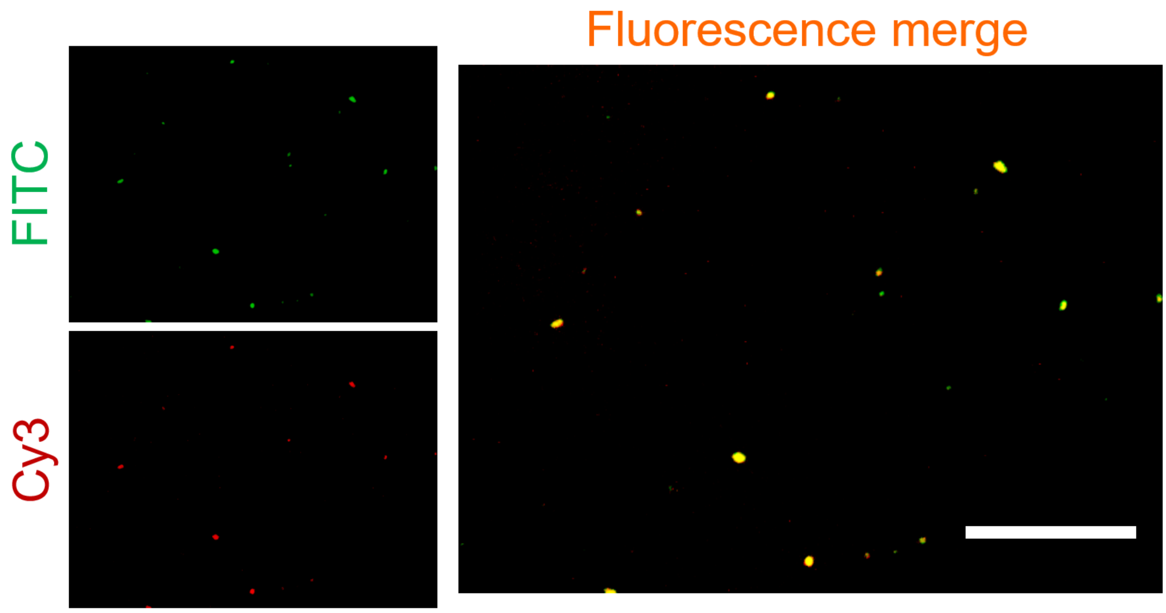

Figure S6. Confocal microscopy image of SOD-Fe^0@Lapa-Z camouflaged with fluorescence-labeled RBC membrane: FITC-labeled SOD-Fe^0@Lapa-Z are shown as green fluorescence, and Cy3-labeled RBC membranes are shown as red fluorescence. The SOD-Fe^0@Lapa-Z and RBC membrane are well fused together after extrusion.

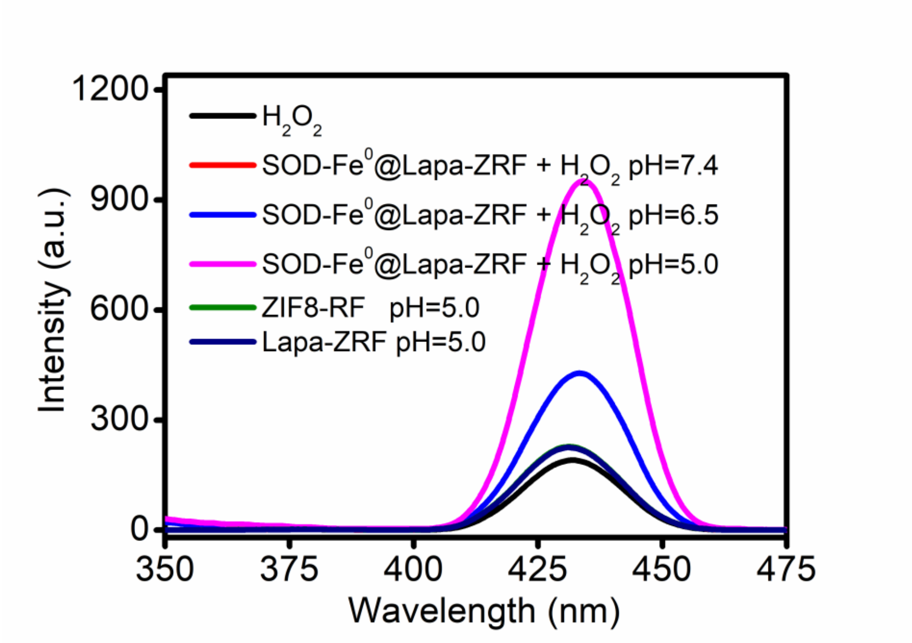

Figure S7. Fluorescence spectra of TPA-OH induced by TPA with the generated ·OH.

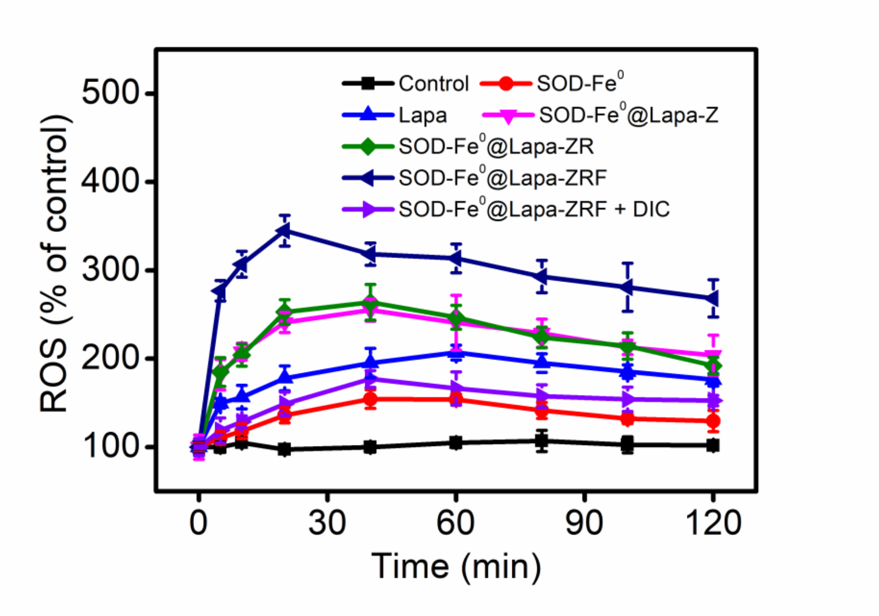

Figure S8. The ROS level changes of 4T1 cells after treatment with different agents without or with dicoumarol. All data represents means ± SD (n = 3).

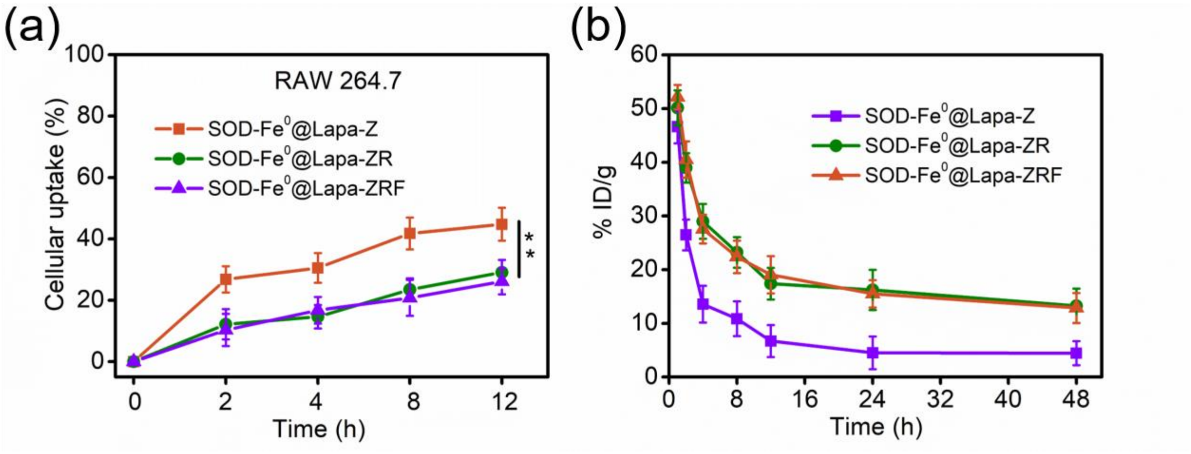

Figure S9. The biocompatibility studies of SOD-Fe^0@Lapa-ZRF. (a) The selectively cellular uptake of SOD-Fe^0@Lapa-ZRF in RAW 264.7 cells. (b) Pharmacokinetic curves of SOD-Fe^0@Lapa-Z, SOD-Fe^0@Lapa-ZR, and SOD-Fe^0@Lapa-ZRF. Data are shown as mean ± SD (n = 3). **$p < 0.01$.

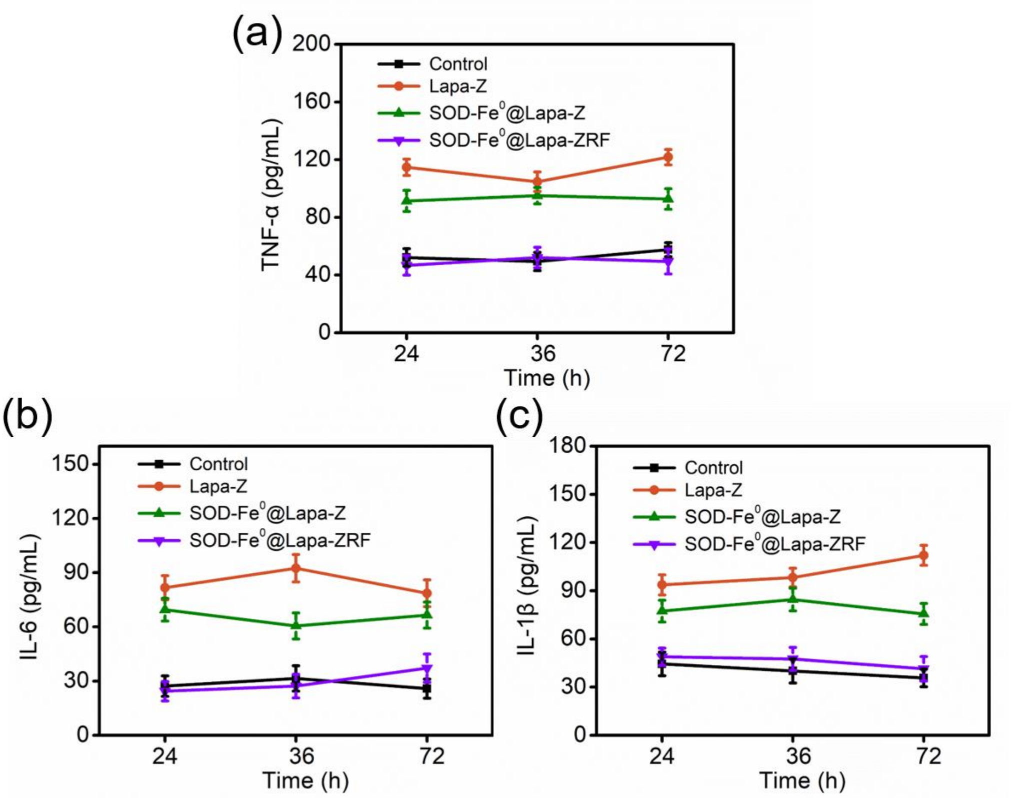

Figure S10. The anti-inflammatory studies of SOD-Fe^0@Lapa-ZRF. ELISA analysis of (a) TNF-α, (b) IL-6, and (c) IL-1β level in serum. Data are shown as mean ± SD (n = 3).

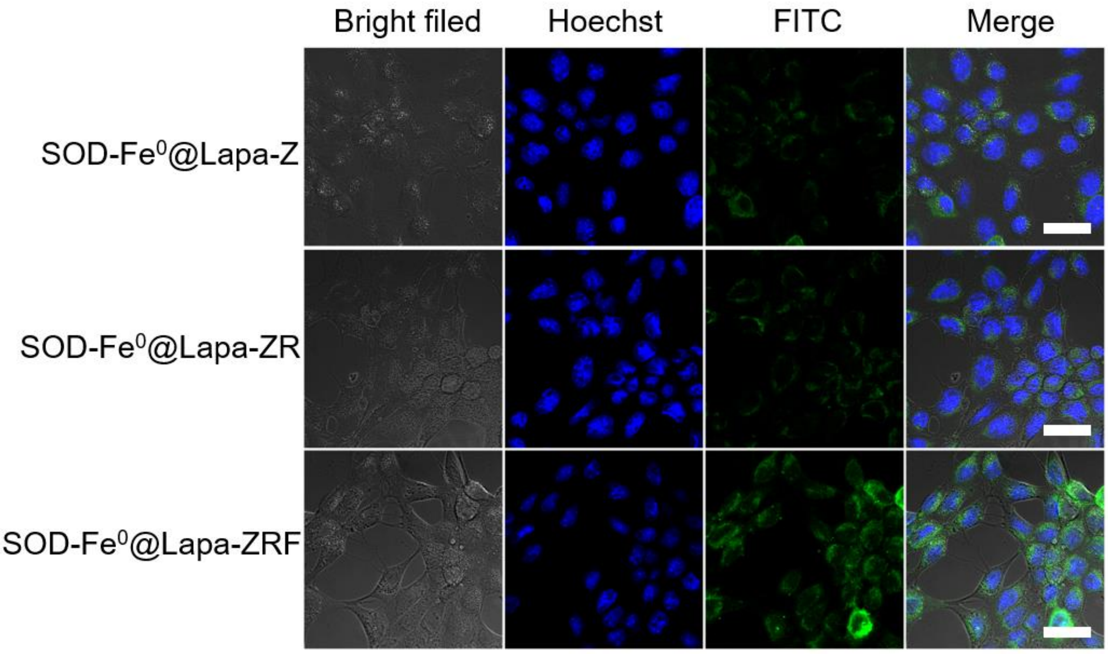

Figure S11. CLSM of 4T1 cells cultured with FITC-loaded SOD-Fe^0@Lapa-Z, SOD-Fe^0@Lapa-ZR, and SOD-Fe^0@Lapa-ZRF. The scale bar is 50 μm.

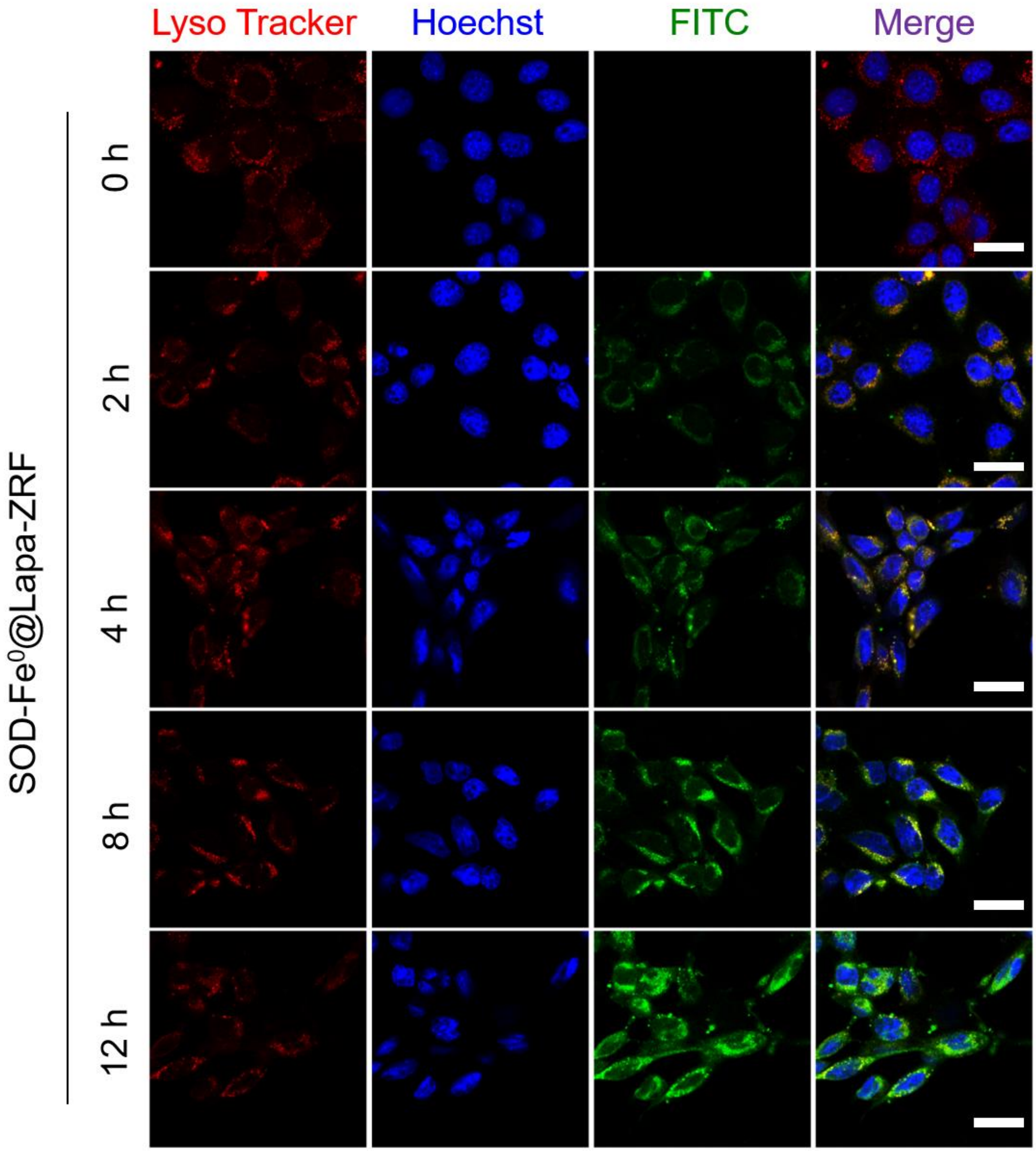

Figure S12. Intracellular trafficking of FITC-loaded SOD-Fe^0@Lapa-ZRF in 4T1 cells for different time. The nuclei and lysosomes were stained with Hoechst 33342 (blue) and LysoTracker (red), respectively. The scale bar is 50 μm.

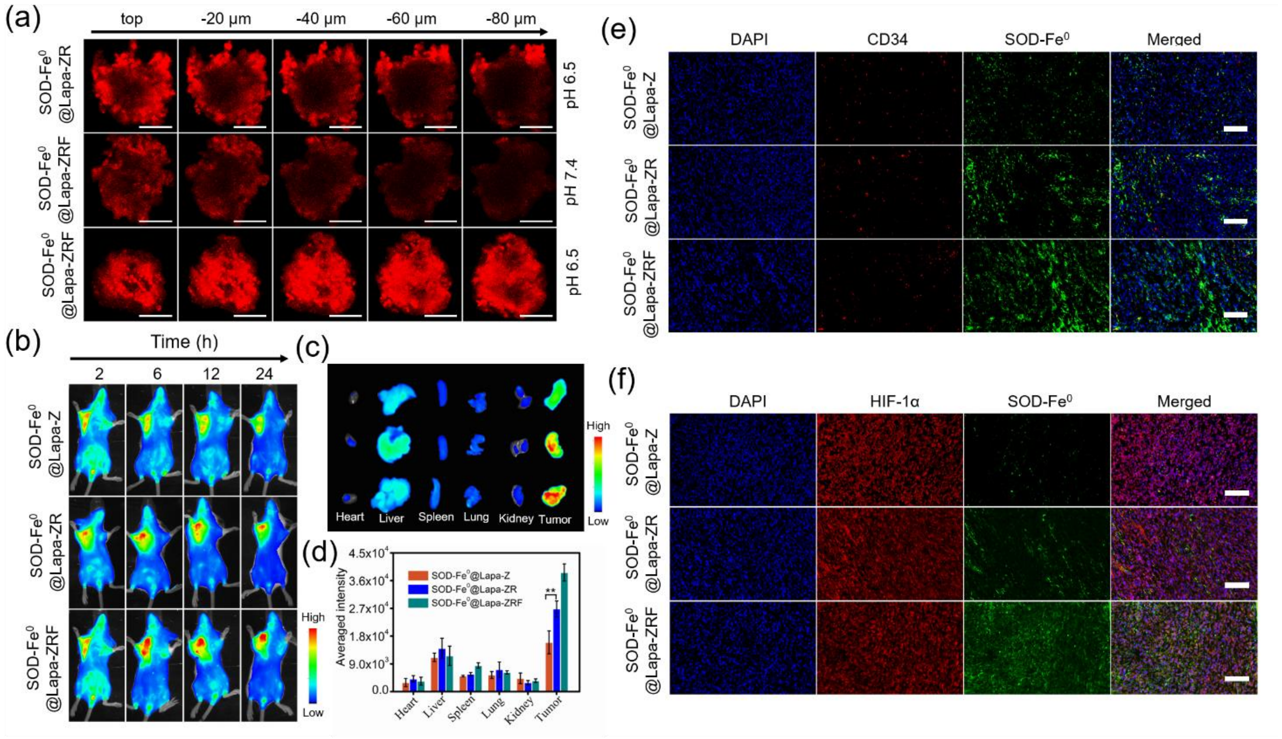

Figure S13. Selectively Tumor targeting and penetration of SOD-Fe^0@Lapa-ZRF. (a) Deep penetration of SOD-Fe^0@Lapa-ZR and SOD-Fe^0@Lapa-ZRF in 4T1 MCTS at pH 7.4 or 6.5. The scale bar is 100 μm. (b) *In vivo* fluorescence imaging of the 4T1 tumor-bearing mice at different points after intravenous injection of SOD-Fe^0@Lapa-Z, SOD-Fe^0@Lapa-ZR, and SOD-Fe^0@Lapa-ZRF. (c) Ex vivo tissue distribution of tumors and major organs at 24 h after nanoparticle administration. (d) ROI analysis of the fluorescence intensities in the main organs collected at 24 h postinjection. $^{**}p < 0.01$. Immunofluorescence images of frozen tumor sections from FITC-labeled SOD-Fe^0@Lapa-Z, SOD-Fe^0@Lapa-ZR, and SOD-Fe^0@Lapa-ZRF treated mouse that was sacrificed 6 h after intratumoral injection. the vascular sparing region (e) and the hypoxic region (f) were shown in red. The scale bar is 50 μm.

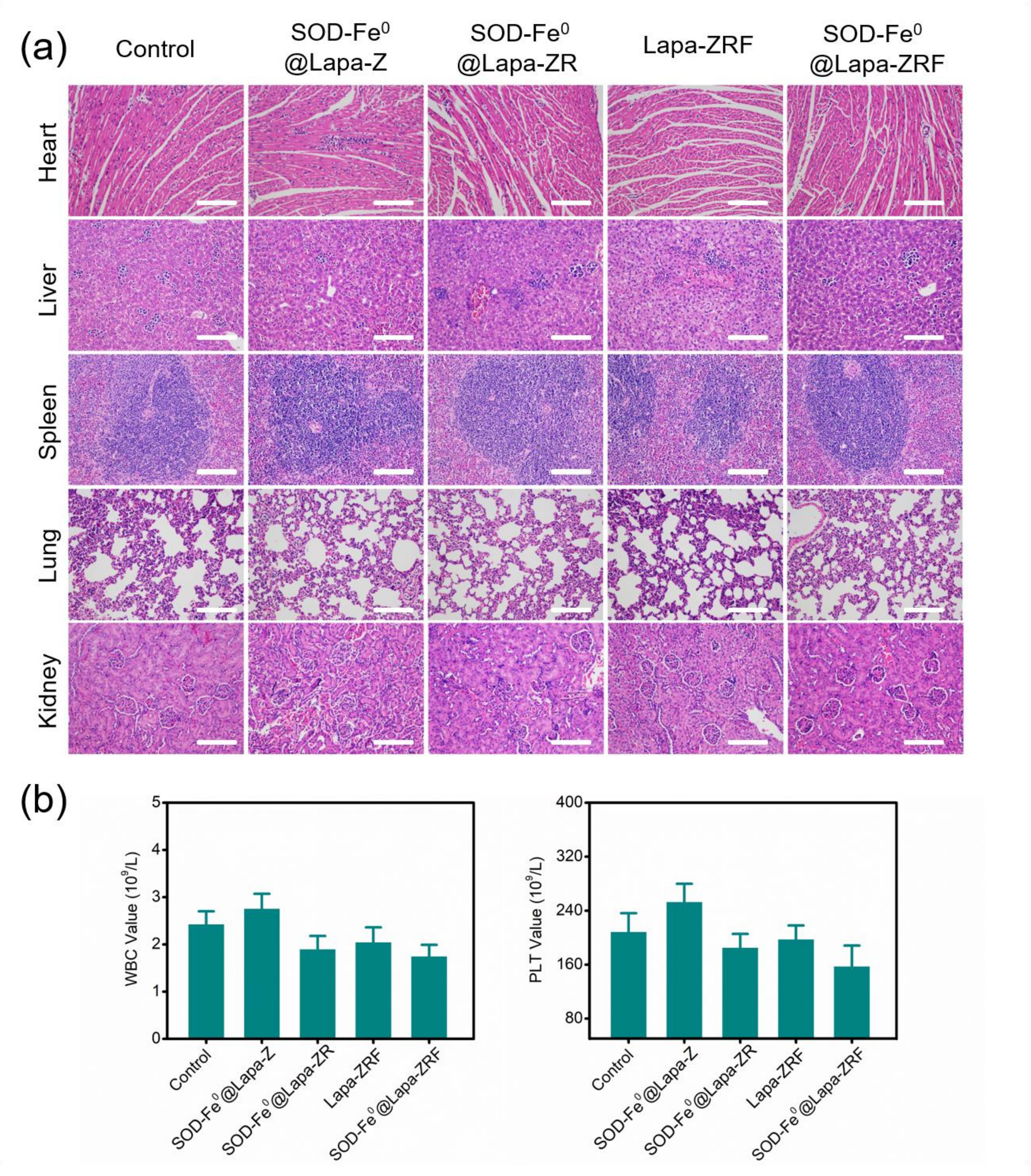

Figure S14. *In vivo* toxicity assessment of SOD-Fe^0@Lapa-ZRF. (a) H&E staining of the major organs from mice after treated with different agents. The scale bar is 100 µm. (b) Blood biochemistry data including numbers of WBC, PLT. Data are shown as mean ± SD (n = 3).
